# Highly Efficient MOF-Driven Silver Subnanometer Clusters for the Catalytic Buchner Ring Expansion Reaction

**DOI:** 10.1021/acs.inorgchem.2c01508

**Published:** 2022-07-21

**Authors:** Estefanía Tiburcio, Yongkun Zheng, Marta Mon, Nuria Martín, Jesús Ferrando−Soria, Donatella Armentano, Antonio Leyva−Pérez, Emilio Pardo

**Affiliations:** † Departamento de Química Inorgánica, Instituto de Ciencia Molecular (ICMOL), Universidad de Valencia, Valencia 46980, Spain; ‡ Instituto de Tecnología Química (UPV−CSIC), Universidad Politècnica de València−Consejo Superior de Investigaciones Científicas, Avda. de los Naranjos s/n, Valencia 46022, Spain; § Dipartimento di Chimica e Tecnologie Chimiche, Università della Calabria, Rende, Cosenza87036, Italy

## Abstract

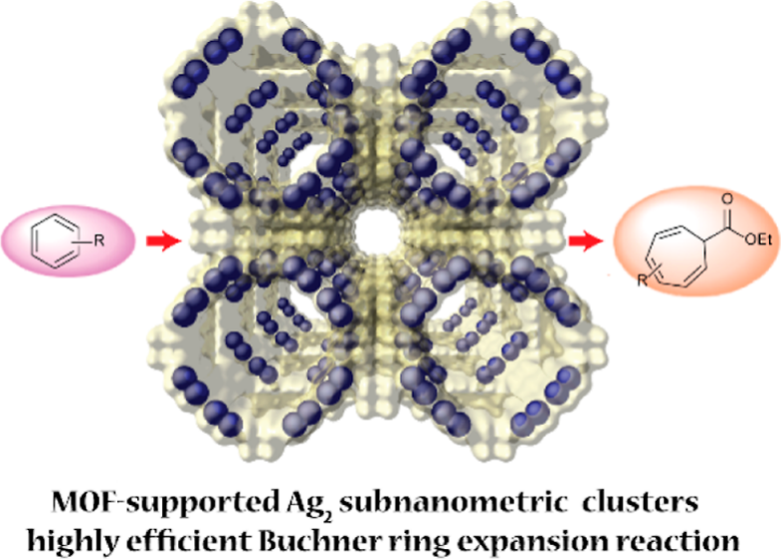

The preparation of novel efficient catalysts—that could be applicable in industrially important chemical processes—has attracted great interest. Small subnanometer metal clusters can exhibit outstanding catalytic capabilities, and thus, research efforts have been devoted, recently, to synthesize novel catalysts bearing such active sites. Here, we report the gram-scale preparation of Ag_2_^0^ subnanometer clusters within the channels of a highly crystalline three-dimensional anionic metal–organic framework, with the formula [Ag_2_^0^]@Ag^I^_2_Na^I^_2_{Ni^II^_4_[Cu^II^_2_(Me_3_mpba)_2_]_3_}·48H_2_O [Me_3_mpba^4–^ = *N,N*′-2,4,6-trimethyl-1,3-phenylenebis(oxamate)]. The resulting crystalline solid catalyst—fully characterized with the help of single-crystal X-ray diffraction—exhibits high catalytic activity for the catalytic Buchner ring expansion reaction.

## Abstract 2

The present work describes the MOF-driven preparation of well-defined ligand-free Ag_2_^0^ nanoclusters, which are efficiently stabilized by the MOF channels. The small Ag_2_^0^ nanoclusters, prepared in the gram scale, are used as efficient and recoverable catalysts for the Buchner ring expansion reaction. These results expand the toolkit of readily affordable Ag species for heterogeneous catalysis in organic synthesis.

## Introduction

The preparation, stabilization, and characterization of subnanometer metal clusters (SNMCs) has been a main challenge for chemists during the last years.^1−7^ Indeed, the preparation of such ultrasmall entities is highly complex and often requires, for instance, the use of stabilizing blocking ligands^8^ that prevent their agglomeration into larger metal nanoparticles (MNPs) but, in turn, may worsen their catalytic properties. Moreover, the characterization of such tiny SNMCs is really not an easy task and requires the use of high-resolution microscopy techniques—for example, high-angle annular dark-field–scanning transmission electron microscopy (HAADF–STEM)—whose electron beam can, with time, degrade the sample that is being observed.^9^ However, despite such complications, the outstanding medical, optical, and/or catalytic properties of SNMCs make it well worth the effort.^3,4,10,11^

Focusing on the catalytic properties of SNMCs, stabilizing capping ligands of SNMCs dramatically reduces their catalytic activity as they prevent a proper contact with reactants and trigger their decomposition under reaction conditions. Therefore, it seems clear that “naked” SNMCs, with all metal atoms exposed, are called to offer the best catalytic performances.^12^ In this context, a proper strategy to obtain ligand-free SNMCs consists in supporting these metal species in porous solids such as zeolites or organic porous materials.^13^ More recently, another type of porous materials, the so-called metal–organic frameworks (MOFs),^14^ have emerged as a suitable platform not only to host SNMCs but also to be used as chemical reactors for the in situ chemical synthesis of the SNMCs.^15,16^

MOFs are crystalline porous materials that have attracted significant attention in the past 2 decades due to the myriad applications they can be used in.^17^ Moreover, aiming at encapsulating/synthesizing SNMCs, or even single-atom catalysts,^18−22^ MOFs offer clear advantages compared to other porous materials such as a fine control of the functionalities decorating the channels—which allows us to retain and align metals in specific positions and controlled stoichiometries—and the possibility to use single-crystal X-ray crystallography^23−25^ to unveil the crystal structure of these ultrasmall metal species.

**Figure 1:**
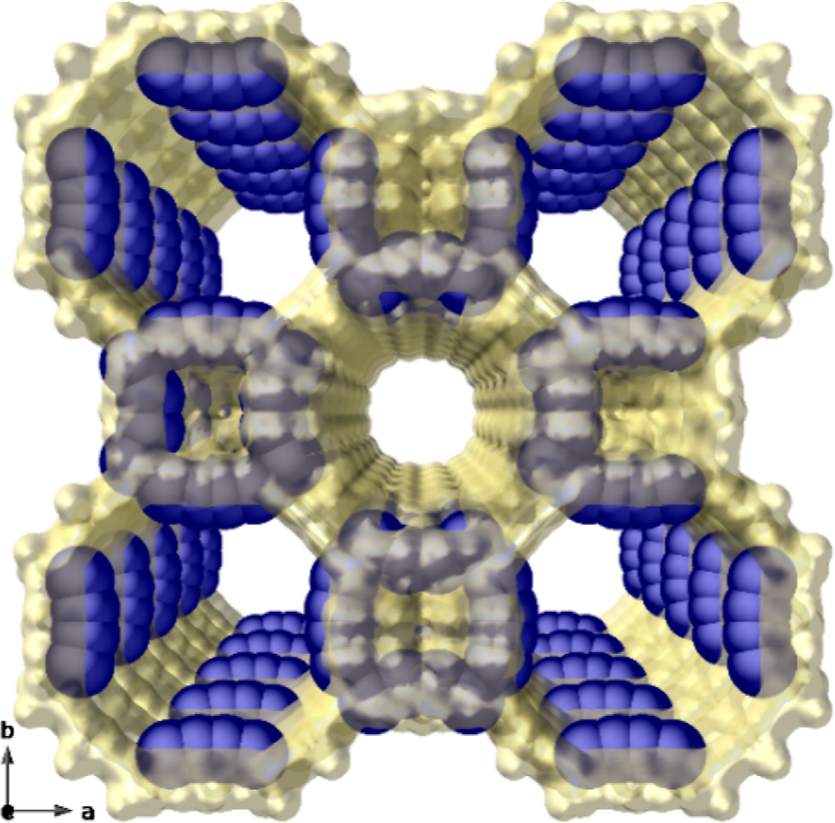
Crystal structure of the previously reported [Pd_4_]_0.5_@Na_3_{Ni^II^_4_[Cu^II^_2_(Me_3_mpba)_2_]_3_}·56H_2_O MOF.^21^ Atoms constituting the framework and Pd atoms are represented by light yellow and blue spheres, respectively.

In this context, we recently used,^21^ as a chemical reactor, a highly robust anionic three-dimensional MOF, with the formula Ni^II^_2_{Ni^II^_4_[Cu^II^_2_(Me_3_mpba)_2_]_3_}·54H_2_O [Me_3_mpba^4–^ = *N,N*′-2,4,6-trimethyl-1,3-phenylenebis(oxamate)], for the MOF-driven preparation of ligand-free tetranuclear [Pd_4_]^2+^ clusters after two consecutive post-synthetic steps consisting of first replacing the Ni^2+^ cations hosted within its channels by Pd^2+^ ones and the concomitant reduction to form the final tetranuclear species within the empty space of the MOF. The resulting host–guest material had the following formula: [Pd_4_]_0.5_@Na_3_{Ni^II^_4_[Cu^II^_2_(Me_3_mpba)_2_]_3_}·56H_2_O (Figure 1). Overall, the anionic nature of the MOF allowed a fine control of the number of inserted Pd^2+^ cations in the first step, which were occupying specific positions by interacting with the carboxylate oxygens from the network. Then, the confined space, as well as the mentioned controlled stoichiometry, allowed the formation of the small [Pd_4_]^2+^ clusters, homogeneously distributed within the walls of the MOF (Figure 1). As expected, such naked clusters, possessing all four metal atoms outwardly exposed, exhibited outstanding catalytic activity, outperforming state-of-the-art metal catalysts in carbene-mediated reactions, also showing high yields (>90%) and turnover numbers (up to 100,000). However, considering the high cost of palladium, it would be highly desirable to achieve highly performing SNMCs with lower prices.

## Results and Discussion

**Figure 2:**
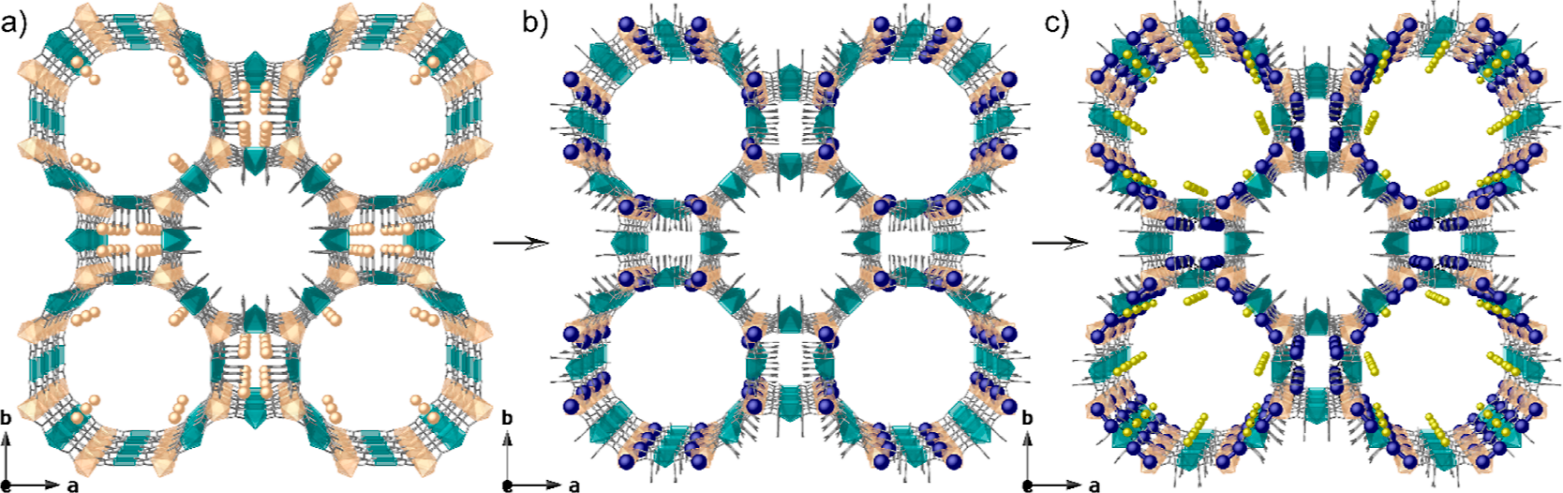
Design approach showing the crystal structures of **1** (a), **2** (b), and **3** (c) showing the two-step post-synthetic process consisting of the exchange of the Ni^II^ cations in the pores of 1 by Ag^I^ ones to yield **2** and the reduction process to form the Ag_2_^0^ clusters in **3**. Virtual diameters of larger octagonal pores are 2.0 nm for MOFs **1**–**3**, respectively. Copper and nickel atoms from the network are represented by cyan and orange polyhedra, respectively, whereas organic ligands are depicted as gray sticks. Orange, yellow, and blue spheres represent Ni, Na, and Ag atoms, respectively.

Herein, aiming at expanding these results to more affordable metals, we report the two-step post-synthetic preparation of Ag_2_^0^ nanoclusters using the same MOF Ni^II^_2_{Ni^II^_4_[Cu^II^_2_(Me_3_mpba)_2_]_3_}·54H_2_O (**1**) as the host matrix (Figure 2a). First, nickel(II) cations, located in the pores of **1**, are exchanged by Ag^+^ ones, yielding the novel compound Ag^I^_4_{Ni^II^_4_[Cu^II^_2_(Me_3_mpba)_2_]_3_}·51H_2_O (**2**) (Figure 2b). Then, after introducing NaBH_4_, the reduction process occurs to give the final compound [Ag_2_^0^]@Ag^I^_2_Na^I^_2_{Ni^II^_4_[Cu^II^_2_(Me_3_mpba)_2_]_3_}·48H_2_O (**3**) (Figure 2c). The whole process could be followed by single-crystal X-ray diffraction (SCXRD), unveiling certain details about the nanocluster formation, which constitutes one of the very few examples of MOF-hosted silver subnanometer clusters^26^ and the first whose crystal structure could be elucidated.

The nature of the final hybrid material, **3**, containing Ag_2_ clusters (together with unreduced Ag^+^ ions) has been further confirmed by the combination of a variety of techniques including inductively coupled plasm–mass spectrometry (ICP–MS) (Table S1, Supporting Information), elemental mapping, powder X-ray diffraction (PXRD), thermogravimetric analysis (TGA), X-ray photoelectron spectroscopy (XPS), and scanning electron microscopy (SEM). The N_2_ adsorption isotherms at 77 K confirmed the permanent porosity of **2** and **3** (see below). Finally, as previously mentioned, SCXRD with synchrotron radiation allowed the resolution of the crystal structure of **2** and **3** (Table S2, Supporting Information) and the observation of ultrasmall silver dinuclear entities and surroundings within the solid in **3** (Figures 2 and S1).

**Figure 3:**
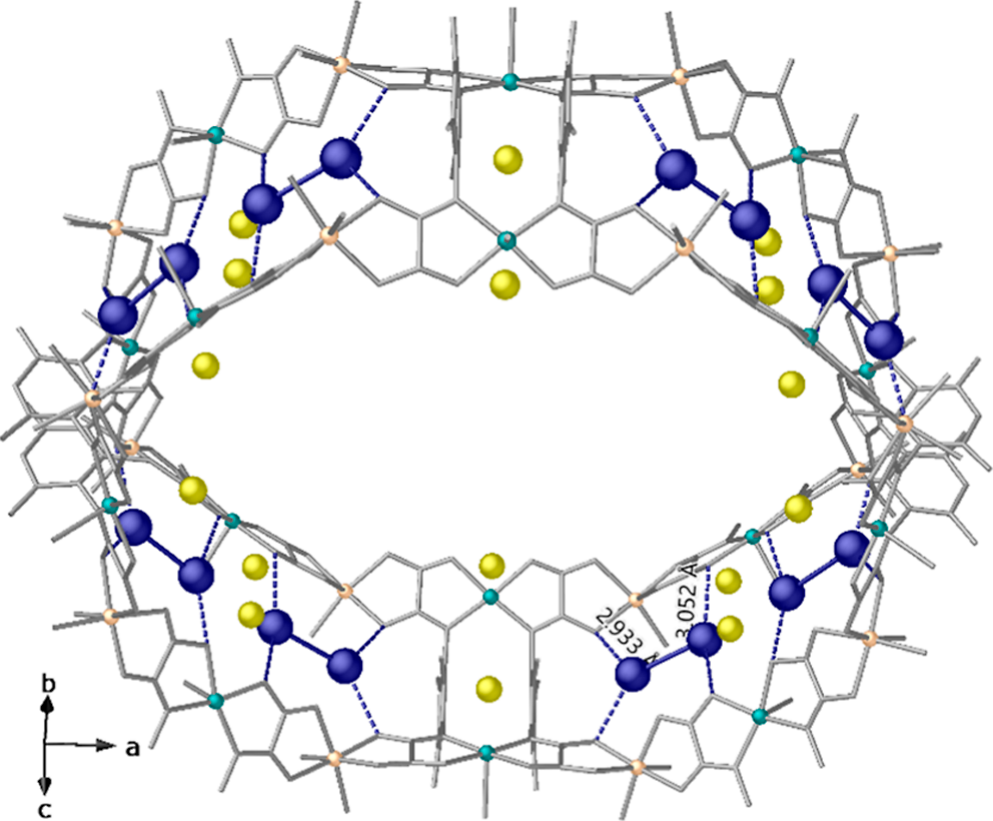
One single channel of **3**, showing supramolecular interactions involving oxamate ligands of the network (distances are reported in angstroms).

The anionic Ni^II^_4_Cu^II^_6_ open-framework structures in both **2** and **3** are isoreticular and crystallize in the *P*4/*mmm* space group of the tetragonal system. Compound **2** exhibits the Ag^+^ cations situated within the walls of the hydrophilic octagonal pores (virtual diameter of 2.0 nm), where they are stabilized by noncovalent interactions involving oxamate oxygen atoms [Ag^+^···O_oxamate_ of 2.72(1)–2.79(1) Å], with no evidence of previously Ni^2+^ cations of **1**, thus indicating that they are completely exchanged by Ag^+^ ones (Figure 2b). Ag^+^ ion surroundings unveil interacting oxygen atoms likely belonging to nitrate anions (the whole fragments were not found from the ΔF map, see Supporting Information, Figures S1–S3) or solvent water molecules [Ag^+^···O distance range of 2.38(3)–2.56(3) Å], together with Ag^+^···Ag^+^ with a separation of 2.74(2) Å, which is shorter than the van der Waals contact distance, and they might be considered as precursors of the Ag_2_^0^ dimers observed in **3**. On the other hand, the crystal structure of **3** reveals the stabilization provided by the nano-confined space of the MOF on the as-synthesized Ag_2_^0^ dimers, constricted into the walls of the hydrophilic octagonal channels of the anionic Ni_4_^2+^Cu_6_^2+^ open-framework net (Figures 2c, S4, and S5), together with unreduced Ag^+^···Ag^+^ dimers in smaller square pores (Figures 2c and S5). Further hydrated charge-counterbalancing alkali Na^+^ cations are retained in the preferential cationic sites, which stabilize the final material, showing an outstanding robustness (Figures S4 and S5). The poorer accessibility to the small square pores for solvated NaBH_4_ is most likely the reason for still unreduced Ag^+^···Ag^+^ dimers [blocked by Ag^+^···O_oxamate_ interactions at a distance of 2.84(1) Å] (Figure S5). Figure 3 shows that Ag_2_^0^ dimers [intradimer Ag···Ag distance of 3.19(1) Å] are well-fixed and stabilized inside the walls of the largest pores of the network by means of supramolecular interactions involving oxamate ligands [Ag···O_oxamate_ distance range of 2.93(1)–3.05(1) Å] and very weak connections with solvent molecules [Ag^0^···O_water_ distance of 3.25(1) Å].

SEM coupled with energy-dispersive X-ray spectroscopy (EDX) measurements of **2** and **3** are given in Figures S6 and S7. EDX elemental mappings for Cu, Ni, Ag, and Na (**3**) elements show a heterogeneous spatial distribution of Ag atoms always located next to Cu and Ni atoms. Moreover, aberration-corrected HAADF–STEM (AC-HAADF–STEM) images are shown in Figure S8. They allow a direct visualization of both Ag_2_ dimers together with Ag_1_ species—most likely to silver atoms residing in smaller square channels.

TGA of **2** and **3** (Figure S9) established the solvent contents for both materials, which are reflected in their chemical formulas. PXRD patterns of **2** and **3** (Figure S10) indicate that the bulk samples are crystalline and pure, with no typical peaks of Ag^0^ nanoparticles. Indeed, experimental diffraction patterns of **2** and **3** are identical to the theoretical ones extracted from the SCXRD data. XPS spectra of compounds **2** and **3** are depicted in Figure S11. For **2**, only possessing Ag^+^ cations, two bands at 367.6 and 373.6 eV, ascribed to Ag 3d_5/2_ and Ag 3d_3/2_ binding energies, respectively,^27^ are observed (Figure S11a). In turn, for MOF **3**—where SCXRD and elemental analyses suggest that Ag^+^ cations and Ag_2_^0^ nanoclusters coexist—apart from the same Ag 3d_5/2_ and Ag 3d_3/2_ bands at 367.6 and 373.6 eV, respectively, indicative of Ag^+^, two additional peaks at 368.4 and 374.4 eV can be observed, which are attributed to reduced Ag^0^ atoms, with a 1:1 ratio respect to Ag^+^ (Figure S11b). CO-probe diffuse reflectance infrared Fourier transform spectroscopy (DRIFTS) was conducted on MOF **3**, run at 77 K to avoid any in situ reduction of Ag^+^ and to observe potential Ag^0^–CO species (Figure S12). The results show three main peaks, one at 1938 cm^–1^, consistent with CO bridged-bonded to Ag^0^ atoms,^28^ a second at 2059 cm^–1^, attributable to Ag(CO)^+^ species,^29^ and a last peak at 2043 cm^–1^, corresponding to free CO, after saturation. It is known that the adsorption of CO on Ag^0^ is lower than Ag^+^;^30^ thus, the lower intensity of the former makes sense and could very well correspond to a 1:1 ratio between Ag oxidation states. These results strongly support that 50% of Ag^+^ present in **2** are reduced by NaBH_4_ forming Ag_2_^0^ nanoclusters, whereas 50% of Ag^+^ cations remain untouched, occupying inaccessible sheltered interstitial positions where the reducing agent cannot accede (see the structural description). The N_2_ and CO_2_ adsorption isotherms for **1**–**3** confirm their permanent porosity (Figures S13 and S14). N_2_ adsorption isotherms for **1**–**3**, with calculated Brunauer–Emmett–Teller^31^ surface areas of 974, 1013, and 625 m^2^/g, respectively, indicate a very similar permanent porosity for **1** and **3**, which is in agreement with their identical estimated virtual diameters of 2.0 nm. In turn, MOF **2** exhibits lower N_2_ adsorption despite having the same virtual diameter (2.0 nm), which could be due to a partial collapse of the structure upon solvent evacuation treatment. Remarkably, CO_2_ adsorption isotherms show a 66% uptake increase for **3**, suggesting quadrupole interactions between CO_2_ molecules and Na^+^ cations.

**Figure 4:**
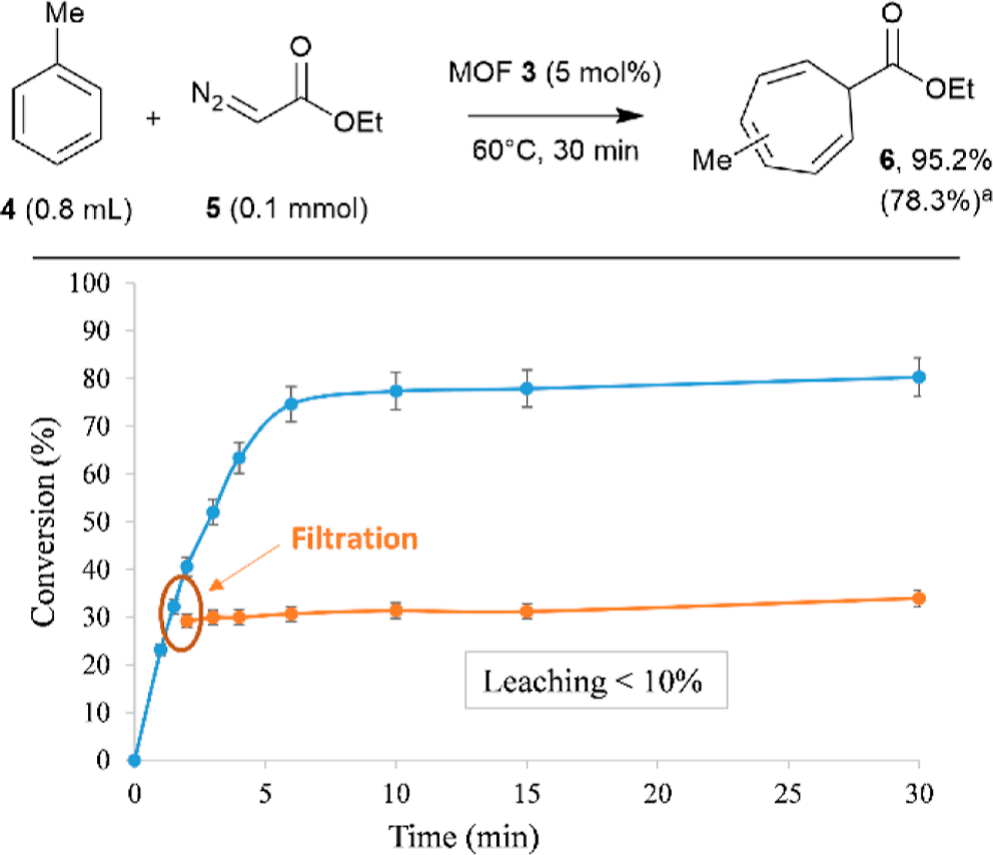
Top: results for the Buchner ring expansion reaction between toluene **4** and ethyl diazoacetate **5** catalyzed by MOF **3**. Bottom: Hot filtration test after adding **5** at once, error bars account for 5% uncertainty. (a) GC yield after syringe pump addition of 5, with the result after the addition of **5** at once given in parentheses; the result with Rh_2_(OAc)_4_ as a catalyst (5 mol %) is 63%.

The Buchner ring expansion reaction was attempted with catalytic amounts of **3**. The results show that the reaction between toluene (**4**) and ethyl diazoacetate (**5**) (EDA) proceeds rapidly (30 min) in a very high yield, under standard reaction conditions (Figure 4 top).^21,32^ Blank experiments without any catalyst gave a 8% conversion, and the use of MOFs **1** and **2** as catalysts showed the lower catalytic activity of these MOFs than that of **3**, with a 3 times lower initial rate for the former (Figure S15). Commercial Ag NPs on alumina only gave a 16% conversion, and remarkably, the state-of-the art catalyst for this reaction, that is, Rh_2_(OAc)_4_, gave a lower result than MOF **3** in this study, under identical reaction conditions (63% after the addition of **5** at once). An optimum >95% yield of product **6** was obtained after maintaining a low concentration of **5** during the reaction, which was achieved by adding a solution of **5** (in dichloromethane) into the reaction mixture using a syringe pump, instead of adding it at once. Otherwise, the unwanted dimerization reaction of **5** occurs. It is worth noting here that product **6** corresponds to the typical mixture of cycloheptatriene isomers, in accordance with previous results.^21,32^ A hot filtration test, where the solid MOF catalyst **3** is removed from the reaction mixture at the reaction temperature (60 °C) at an early conversion (∼30%), shows that the catalytic active species are not present in solution within the experimental error (<10%, Figure 4 bottom), which supports the relative stability of the solid catalyst. In accordance with this result, MOF **3** can be recovered at the end of the reaction by centrifugation, washed, and reused six times, maintaining a good catalytic activity (Figure S16). However, the catalytic yield of MOF **3** decreased to 30% after six uses, which could be due to the progressive (although minor) leaching of active species during the reaction.

The Buchner ring expansion reaction catalyzed by **3** could be expanded to other aromatic substrates (Table 1). Halogenated (products **13**, **14**, and **17**), cyano (product **15**), methoxy (product **16**), and ortho-disubstituted (product **18**) aromatic compounds react with **5** in good to excellent yields and still in short reaction times (<2 h). These results should be remarked upon as this is difficult to find in the open literature Ag-catalyzed Buchner ring expansion reactions.^32−35^ The slightly lower results obtained for the bigger substrates **11** and **12** can be explained by the size discrimination associated to the microporous structure of MOF **3**. To check this, 1,3,5-triisopropylbenzene **19** was tested as a substrate for the reaction, and product **20** was not found (Figure S17). Besides, diffusion tests with varying stirring speeds confirmed that the initial reaction rate is dependent on the stirring, which confirms that the reaction occurs inside the MOF’s channels (Figure S18). Indeed, 1,3,5-triisopropylbenzene **19** reacted when the Rh_2_(OAc)_4_ salt was used as a catalyst (3 mol %, 69% yield, Figure S17). Therefore, for MOF- or zeolite-stabilized Pd clusters,^21,36^ the formation of Ag_2_ clusters in micro-structured solids enables not only the catalytic use of this metal in this complex organic synthetic reaction but also its recovery and reuse.^36,37^ Interstitial Ag^+^ cations must be excluded as catalytically active species since reagents cannot access these sites; besides, leaching does not occur under the reaction conditions employed. Finally, the integrity of MOF **3** is ensured by PXRD (Figure S19) and XPS (Figure S20) after catalytic experiments.

**Table 1:**
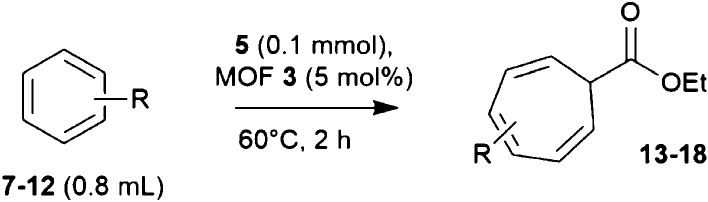
Results for the Buchner Ring Expansion Reaction between Different Aromatics 7–12 and 5, Catalyzed by MOF 3^a^

entry	aryl substrate	substituent(s)	product	yield (%)^a^
1	**7**	Cl	**13**	66.5
2	**8**	Br	**14**	64.9
3	**9**	CN	**15**	92.8
4	**10**	OMe	**16**	82.6
5	**11**	CH_2_Br	**17**	50.2
6	**12**	Me-*ortho*-F	**18**	72.7

a GC yields after syringe pump addition of 5.

## Conclusions

In conclusion, ligand-free Ag_2_^0^ clusters have been prepared, stabilized and characterized within an MOF and used as efficient and recoverable catalysts for the Buchner ring expansion reaction. These results expand the toolkit of readily affordable Ag species for heterogeneous catalysis in organic synthesis.

## Experimental Section

### Preparation of Ag^I^_4_{Ni^II^_4_[Cu^II^_2_(Me_3_mpba)_2_]_3_}·51H_2_O (**2**)

Well-formed deep green prisms of **2**, which were suitable for XRD, were obtained by immersing crystals of **1** (ca. 0.0015 mmol) for 48 h in 5 mL of a AgNO_3_ aqueous solution (0.004 mmol), which was replaced three times. A multigram scale procedure was also carried out by using the same synthetic procedure but with greater amounts of both, a powder sample of compound **1** (*ca.* 20 g, 5.8 mmol) and AgNO_3_ (2.38 g, 14.0 mmol), with the same successful results and a very high yield (20.33 g, 96%). Anal.: calcd (%) for Cu_6_Ni_4_Ag_4_C_78_H_162_N_12_O_87_ (3707.7): C, 25.27; H, 4.40; N, 4.53. Found: C, 25.34; H, 4.37; N, 4.59. IR (KBr): ν = 3008, 2961 and 2926 cm^–1^ (C–H), 1601 cm^–1^ (C=O).

### Preparation of [Ag_2_]Ag^I^_2_Na^I^_2_{Ni^II^_4_[Cu^II^_2_(Me_3_mpba)_2_]_3_}·48H_2_O (**3**)

Both crystals (ca. 5 mg) and a powder polycrystalline sample of **2** (ca. 10 g) were suspended in 50 mL of a H_2_O/CH_3_OH (1:2) solution, to which an excess of NaBH_4_, divided in 26 fractions (each fraction consisting of 1 mol of NaBH_4_ per mole of **2** to give a final NaBH_4_/MOF molar ratio of 26 or a NaBH_4_/Ag atom molar ratio of 13, which is the same), was added progressively in the space of 72 h. After each addition, the mixture was allowed to react for 1.5 h. After this period, samples were gently washed with a H_2_O/CH_3_OH solution and filtered on paper, giving high yields (*ca.* 98%). Anal. Calcd (%) for Cu_6_Ni_4_Ag_4_Na_2_C_78_H_156_N_12_O_84_ (3699.61): C, 25.32; H, 4.25; N, 4.54. Found: C, 25.28; H, 4.17; N, 4.59. IR (KBr): ν = 3011, 2971 and 2928 cm^–1^ (C–H), 1605 cm^–1^ (C=O).

### Gas Adsorption

The N_2_ and CO_2_ adsorption–desorption isotherms at 77 and 273 K were obtained on polycrystalline samples of **2** and **3** using a BELSORP-mini-X instrument. Samples were first activated with methanol and then evacuated at 348 K during 19 h under 10^–6^ Torr prior to their analysis.

### Microscopy Measurements

SEM elemental analysis was carried out for **2** and **3** using a HITACHI S-4800 electron microscope coupled with an EDX detector. Data were analyzed using QUANTAX 400.

HAADF–STEM characterization for **3** was performed using an HAADF-FEI-TITAN G2 electron microscope. 5 mg of the material was re-dispersed in 1 mL of absolute EtOH. Carbon-reinforced copper grids (200 mesh) were submerged into the suspension 30 times and then allowed to dry on air for 24 h.

### PXRD Measurements

Polycrystalline samples of **2** and **3** were introduced into 0.5 mm borosilicate capillaries prior to being mounted and aligned on an Empyrean PANalytical powder diffractometer, using Cu Kα radiation (λ = 1.54056 Å). For each sample, five repeated measurements were collected at room temperature (2θ = 2–60°) and merged in a single diffractogram.

### XPS Measurements

Samples of **2** and **3** were prepared by sticking, without sieving, the samples onto a molybdenum plate using a scotch tape film, followed by air-drying. Measurements were performed on a K-Alpha XPS system using a monochromatic Al K(alpha) source (1486.6 eV). As an internal reference for the peak positions in the XPS spectra, the C 1s peak has been set at 284.8 eV.

### DRIFTS of Adsorbed CO

DRIFTS using CO as a probe molecule was used to evaluate the electronic properties of MOF **3**. The experiments have been carried out in a homemade IR cell able to work in the high and low (77 K) temperature ranges. Prior to CO adsorption experiments, the sample was evacuated at 298 K under vacuum (10^–6^ mbar) for 1 h. CO adsorption experiments were performed at 77 K in the 0.2–20 mbar range. Spectra were recorded once complete coverage of CO at the specified CO partial pressure was achieved. Deconvolution of the IR spectra has been performed in the Origin software using Gaussian curves where the full width at half-maximum of the individual bands has been taken as a constant. The peak areas are normalized to the sample weight.

### X-ray Crystallographic Data Collection and Structural Refinement

Crystals of **2** and **3** with ca. 0.06 × 0.08 × 0.08 and 0.08 × 0.12 × 0.12 mm dimensions, respectively, were selected and mounted on a MITIGEN holder in Paratone oil and very quickly placed on a liquid nitrogen stream cooled at 90 K to avoid the possible degradation upon dehydration. Diffraction data for **2**–**3** were collected using synchrotron at the I19 beamline of the DIAMOND at λ = 0.6889 Å. Crystallographic details can be found in the Supporting Information

### General Catalytic Reaction Procedure

MOF **3** (9.5 mg, 10 mol % Ag) was weighed in a 2 mL vial with a magnetic stirrer, and the aromatic substrate (0.8 mL) was added. Then, the vial was placed in a pre-heated oil batch at 60 °C, and ethyl diazoacetate **5** (0.1 mmol) was added, either at once or using a syringe pump (solution in dichloromethane). The mixture was allowed to react for 0.5–2 h. After the reaction was complete, filtration was carried out to separate the solid catalyst. The reaction mixture was analyzed by gas chromatography (GC) and GC–MS. Further details can be found in the Supporting Information.

## Supporting Information Available

Experimental preparation, analytical characterization; selected data from SEM/EDX and ICP–MS analyses; summary of crystallographic data of **2** and **3**; perspective view along the *c* crystallographic axis of **2** and **3**; details along *c* and *a* crystallographic axes of a single octagonal pore in **2**; one single channel of **2** showing supramolecular interactions involving oxamate ligands of the network stabilizing Ag^+^ dimers; details along *c* and *a* crystallographic axes of a single octagonal pore in **2**; backscattered SEM images of **2** and **3** and corresponding EDX mapping for Cu, Ni, and Ag elements; AC-HAADF–STEM image of MOF 3 showing the presence of both Ag single atoms and Ag_2_ dimers; TGA curves of **2** and **3** under a dry nitrogen atmosphere; theoretical and experimental PXRD pattern profiles of **2** and **3**; XPS spectra of **2** and **3**; CO-DRIFTS spectra of MOF **3** with different CO doses; nitrogen sorption and desorption isotherms for the activated compounds **1**, **2**, and **3**; CO_2_ sorption and desorption isotherms for the activated compounds **1**, **2**, and **3**; kinetics for the Buchner ring expansion reaction between toluene 4 and ethyl diazoacetate 5 catalyzed by MOFs **1** and **3**; catalytic reuses for the Buchner ring expansion reaction between toluene 4 and ethyl diazoacetate 5 catalyzed by MOF 3; Buchner ring expansion reaction between mesitylene 18 and ethyl diazoacetate 5 catalyzed by either MOF 3 or the Rh_2_(OAc)_4_ salt; kinetics for the Buchner ring expansion reaction between toluene 4 and ethyl diazoacetate 5 catalyzed by MOF **3** under increasing stirring speeds and the corresponding initial rate–stirring rate correlation; experimental PXRD pattern of **3** after catalytic experiments; and XPS spectra of **3** after catalytic experiments CCDC reference numbers: 2155455 (**2**) and 2155456 (**3**) (PDF)
