# Supplementary material for: Highly Efficient MOF-Driven Silver Subnanometer Clusters for the Catalytic Buchner Ring Expansion Reaction
Source: Inorg Chem. 2022 Jul 21;61(30):11796–802. doi: 10.1021/acs.inorgchem.2c01508 (PMC9380725; doi:10.1021/acs.inorgchem.2c01508)
Supplement: Supplementary file 1 — ic2c01508_si_001.pdf [file ic2c01508_si_001.pdf]

Highly Efficient MOF-Driven Silver Subnanometer Clusters for the Catalytic Buchner Ring Expansion Reaction

Estefanía Tiburcio,[†,◊] Yongkun Zheng,[‡,◊] Marta Mon,*[,‡] Nuria Martín,*[,†] Jesús Ferrando–Soria,[†] Donatella Armentano,[§] Antonio Leyva–Pérez*[,‡] and Emilio Pardo*[,†]

[†]Departamento de Química Inorgánica, Instituto de Ciencia Molecular (ICMOL), Universidad de Valencia, 46980, Valencia, Spain.

[‡]Instituto de Tecnología Química (UPV–CSIC), Universidad Politècnica de València–Consejo Superior de Investigaciones Científicas, Avda. de los Naranjos s/n, 46022, Valencia, Spain.

[§]Dipartimento di Chimica e Tecnologie Chimiche, Università della Calabria, Rende 87036, Cosenza, Italy.

[◊]These authors have equally contributed to this work

Email: marmoco@itq.upv.es; nuria.martin@uv.es; anleyva@itq.upv.es; emilio.pardo@uv.es

Table of Contents

Experimental Section SI-3

Chemicals SI-3

Physical Techniques SI-4

Catalysis SI-7

Tables S1-S2 SI-8

Figures S1-20 SI-10

Characterization of the products SI-30

Experimental Section

Chemicals

All chemicals were of reagent grade quality. They were purchased from commercial sources and used as received. MOF $Ni^{II}_2\{Ni^{II}_4[Cu^{II}_2(Me_3mpba)_2]_3\} \cdot 54H_2O$ (**1**) as reported earlier.[1] Products were characterised by GC–MS and compared with the given literature.

$Ag^{I}_4\{Ni^{II}_4[Cu^{II}_2(Me_3mpba)_2]_3\} \cdot 51H_2O$ (2): Well–formed deep green prisms of **2**, which were suitable for X–ray diffraction, were obtained by immersing crystals of **1** (ca. 0.0015 mmol) for 48 hours in 5 mL of a $AgNO_3$ aqueous solution (0.004 mmol), which were replaced three times. A multigram scale procedure was also carried out by using the same synthetic procedure but with greater amounts of both, a powder sample of compound 1 (*ca.* 20 g, 5.8 mmol) and $AgNO_3$ (2.38 g, 14.0 mmol), with the same successful results and a very high yield (20.33 g, 96%). Anal.: calcd (%) for $Cu_6Ni_4Ag_4C_{78}H_{162}N_{12}O_{87}$ (3707.7): C, 25.27; H, 4.40; N, 4.53. Found: C, 25.34; H, 4.37; N, 4.59. IR (KBr): ν = 3008, 2961 and 2926 cm^{-1} (C–H), 1601 cm^{-1} (C=O).

$[Ag_2]Ag^{I}_2Na^{I}_2\{Ni^{II}_4[Cu^{II}_2(Me_3mpba)_2]_3\} \cdot 48H_2O$ (3): Both, crystals (ca. 5 mg) and a powder polycrystalline sample of **2** (ca. 10 g), were suspended in 50 mL of a H_2O/CH_3OH (1:2) solution to which an excess of $NaBH_4$, divided in 26 fractions (each fraction consisting of 1 mole of $NaBH_4$ per mole of **2** to give a final $NaBH_4$ / MOF molar ratio of 26 or, which is the same, $NaBH_4$ / Ag atom molar ratio of 13), was added progressively in the space of 72 hours. After each addition, the mixture was allowed to react for 1.5 hour. After this period, samples were gently washed with a H_2O/CH_3OH solution and filtered on paper giving high yields (*ca.* 98%). Anal.: calcd (%) for $Cu_6Ni_4Ag_4Na_2C_{78}H_{156}N_{12}O_{84}$ (3699.61): C, 25.32; H, 4.25; N, 4.54. Found: C, 25.28; H, 4.17; N, 4.59. IR (KBr): ν = 3011, 2971 and 2928 cm^{-1} (C–H), 1605 cm^{-1} (C=O).

[1] Grancha, T.; Ferrando-Soria, J.; Zhou, H.-C.; Gascon, J.; Seoane, B.; Pasán, J.; Fabelo, O.; Julve, M.; Pardo, E. Postsynthetic Improvement of the Physical Properties in a Metal-Organic Framework through a Single Crystal to Single Crystal Transmetallation. *Angew. Chemie Int. Ed.* **2015**, *54*, 6521–6525.

Physical Techniques

Elemental (C, H, N), and ICP-MS analyses were performed at the Microanalytical Service of the Universitat de València. The results are included in the chemical formulas of compounds **2** and **3**. FT–IR spectra were recorded on a Perkin-Elmer 882 spectrophotometer as KBr pellets. The thermogravimetric analysis was performed on crystalline samples under a dry N_2 atmosphere with a Mettler Toledo TGA/STDA 851e thermobalance operating at a heating rate of 10 ºC min^{-1}. Gas chromatographic analyses were performed in an instrument equipped with a 25 cm capillary column of 5% phenylmethylsilicone. *N*-dodecane was used as an external standard. GC/MS analyses were performed on a spectrometer equipped with the same column as the GC and operated under the same conditions.

Gas adsorption: The N_2 and CO_2 adsorption-desorption isotherms at 77 and 273 K, were carried out on policrystalline samples of **2** and **3** with a BELSORP-mini-X instrument. Samples were first activated with methanol and then evacuated at 348 K during 19 hours under 10^{-6} Torr prior to their analysis.

Microscopy measurements: Scanning Electron Microscopy (SEM) elemental analysis was carried out for **2** and **3**, using a HITACHI S-4800 electron microscope coupled with an Energy Dispersive X–ray (EDX) detector. Data was analyzed with QUANTAX 400.

High–Angle Annular Dark–Field Scanning Transmission Electron microscopy (HAADF–STEM) characterization for **3** was done using a HAADF–FEI–TITAN G2 electron microscope. 5 mg of the material was re–dispersed in 1 mL of absolute EtOH. Carbon reinforced copper grids (200 meshs) were submerged into the suspension 30 times and then allowed to dry on air for 24 h.

X–ray Powder Diffraction Measurements: Polycrystalline samples of **2** and **3** were introduced into 0.5 mm borosilicate capillaries prior to being mounted and aligned on a Empyrean PANalytical powder diffractometer, using Cu Kα radiation (λ = 1.54056 Å). For each sample, five repeated measurements were collected at room temperature (2θ = 2–60°) and merged in a single diffractogram.

X–ray photoelectron spectroscopy (XPS) measurements: Samples of **2** and **3** were prepared by sticking, without sieving, the samples onto a molybdenum plate with scotch

tape film, followed by air drying. Measurements were performed on a K-Alpha™ X-ray Photoelectron Spectrometer (XPS) System using a monochromatic Al K(alpha) source (1486.6 eV). As an internal reference for the peak positions in the XPS spectra, the C1s peak has been set at 284.8 eV.

Diffuse reflectance infrared fourier transform spectroscopy (DRIFTS) of adsorbed CO: DRIFTS using CO as a probe molecule was used to evaluate electronic properties of MOF **3**. The experiments have been carried out in a homemade IR cell able to work in the high and low (77 K) temperature range. Prior to CO adsorption experiments, the sample was evacuated at 298 K under vacuum (10^{-6} mbar) for 1 h. CO adsorption experiments were performed at 77 K in the 0.2–20 mbar range. Spectra were recorded once complete coverage of CO at the specified CO partial pressure was achieved. Deconvolution of the IR spectra has been performed in the Origin software using Gaussian curves where the full width at half–maximum (fwhm) of the individual bands has been taken as constant. The peak areas are normalized to the sample weight.

X-ray crystallographic data collection and structure refinement: Crystals of **2**, and **3** with *ca.* 0.06 x 0.08 x 0.08, and 0.08 x 0.12 x 0.12 mm as dimensions were selected and mounted on a MITIGEN holder in Paratone oil and very quickly placed on a liquid nitrogen stream cooled at 90 K to avoid the possible degradation upon dehydration. Diffraction data for **2-3** were collected using synchrotron at I19 beamline of the DIAMOND at λ = 0.6889 Å. The data were processed through CrysAlisPro[2] and xia2[3] software. The structure was solved with the SHELXS structure solution program, using the Patterson method. The model was refined with version 2018/3 of SHELXTL against F^2 on all data by full-matrix least squares.[4]

[2] CrysAlisPro 1.171.38.41, Rigaku Oxford Diffraction, Oxfordshire, England. 2015.

[3] (a) Evans, P. Scaling and Assessment of Data Quality. *Acta Crystallogr. Sect. D Biol. Crystallogr.* **2006**, *62*, 72–82. (b) Evans, P. R.; Murshudov, G. N. How Good Are My Data and What Is the Resolution? *Acta Crystallogr. Sect. D Biol. Crystallogr.* **2013**, *69*, 1204–1214. (c) Winn, M. D.; Ballard, C. C.; Cowtan, K. D.; Dodson, E. J.; Emsley, P.; Evans, P. R.; Keegan, R. M.; Krissinel, E. B.; Leslie, A. G. W.; McCoy, A.; et al. Overview of the CCP 4 Suite and Current Developments. *Acta Crystallogr. Sect. D Biol. Crystallogr.* **2011**, *67*, 235–242. (d) Winter, G. Xia2 : An Expert System for Macromolecular Crystallography Data Reduction. *J. Appl. Crystallogr.* **2010**, *43*, 186–190.

[4] (a) Sheldrick, G. M. Crystal Structure Refinement with SHELXL. *Acta Crystallogr. Sect. C Struct. Chem.* **2015**, *71*, 3–8. (b) Sheldrick, G. M. A Short History of SHELX. *Acta Crystallogr. A.* **2008**, *64*, 112–122.

Crystals of **3**, suitable for X-ray diffraction, were obtained by soaking crystals of **2** (5.0 mg) in a saturated H_2O/CH_3OH (1:2) solution to which an excess of $NaBH_4$, was added progressively in 72 hours. The reduction of the crystals occurs with a crystal-to-crystal transformation. For these reasons it is reasonable to observe a diffraction pattern sometimes affected by expected internal imperfections of the crystals. Furthermore, for the same reasons and due to the huge cell in which compound crystallize and high porosity of the system, it is reasonable to observe a quite poor diffraction power of the samples even if in presence of heavy atoms as copper, nickel and silver. In fact, a completeness of data was obtained at θ_{max} of 23 and 21°, for **2** and **3**, respectively (Table S2) (detected as Alerts A and B in the checkcifs). However, the solution and refinement parameters are suitable, compared with MOFs structures generally reported, thus we are convinced that the structures found are consistent.

While in **2** all non-hydrogen atoms were refined anisotropically except disordered Ag^+ ions and lattice water molecules, in **3** only copper, nickel and their environment has been refined anisotropically in order to maintain a good data/parameter ratio (see Table S2). All attempts to perform improved measurements on a single crystal of **3**, resulting after a crystal-to-crystal transformation and featuring a very huge cell, either at I19 beamline of DIAMOND or in house X-ray facilities failed, due to partial crystal damage / crystal deterioration under reduction conditions. The occupancy factors, of Ag^+ ions have been defined in agreement with SEM results. The use of some C-C bond lengths restrains, SIMU and DELU, during the refinements both in **2** and **3** has been reasonable imposed and related to flexibility of the three–substituted phenyl rings of the Me3mpba ligand that are dynamic components of the frameworks. In the refinement of **2** and **3** crystal structures, some further restrains, to make the refinement more efficient, have been applied. For instance, ADP components have been restrained to be similar to other related atoms, EADP for group of atoms of the guest Ag^+ ions (in **3**) expected to have essentially similar ADPs.

The solvent molecules were disordered but, even if not all the ones detected by TGA analysis, have been somehow modelled. For that reason, in **2** and **3** refinements, the contribution to the diffraction pattern from the disordered water molecules located in the voids was subtracted from the observed data through the SQUEEZE method,

implemented in PLATON.[5] The hydrogen atoms of the ligand in all structure refinement were set in calculated positions and refined as riding atoms whereas for water molecules were neither found nor calculated.

A summary of the crystallographic data and structure refinement for the two compounds is given in Table S2. The somewhat high R values (levels Alert A and B in checkcifs) in **2** and **3** is, most likely, affected by the contribution of the highly disordered solvent to the intensities of the low angle reflections. CCDC 2155455-2155456 for **2** and **3**, respectively.

The final geometrical calculations on free voids and the graphical manipulations were carried out with PLATON[7] implemented in WinGX,[6] and CRYSTAL MAKER[7] programs, respectively.

Catalysis.

General reaction procedure: MOF **3** (9.5 mg, 10 mol% Ag) was weighed in a 2 mL vial with a magnetic stirrer, and the aromatic substrate (0.8 mL) was added. Then, the vial was placed in a pre-heated oil batch at 60 ºC and ethyl diazoacetate **5** (0.1 mmol) was added, either at once or by syringe pump (solution in dichloromethane). The mixture was allowed to react for 0.5-2 h. After the reaction is complete, filtration is carried out to separate the solid catalyst. The reaction mixture was analyzed by GC and GC-MS.

Hot filtration test: Following the general reaction procedure, two parallel reactions were carried out and one of them was rapidly filtrated at the reaction temnperature (60 ºC) after 2 min reaction time (~ 30% conversion). Then, the kinetic profiles for both the solid-containing reaction and the filtrates were assessed and compared.

Reuses: Following the general reaction procedure, the solid catalyts was separated by centrifugation at 4000 r.p.m. during 5 min, washed with dichloromethane (1 mL), separated again and dried. Fresh reactants were placed for a new reaction.

[5] Spek, A. L. Structure Validation in Chemical Crystallography. *Acta Crystallogr. D. Biol. Crystallogr.* **2009**, *65*, 148–155.
[6] Farrugia, L. J. WinGX Suite for Small-Molecule Single-Crystal Crystallography. *J. Appl. Crystallogr.* **1999**, *32*, 837–838.
[7] D. C. Palmer, Crystalmaker, CrystalMaker Software Ltd, Begbroke, Oxfordshire, England. 2014.

Table S1. Selected data from the ICP–MS[a] and SEM/EDX[b] analyses.

		MOF 2		
**Metal**	*% mass*[a]	*Metal stoichiometry*[a]	*% mass*[b]	*Metal stoichiometry*[b]
**Cu**	10.297	6.01	10.17	5.93
**Ni**	6.329	4.00	6.23	3.93
**Ag**	11.715	4.02	11.99	4.12
		**MOF 3**		
**Metal**	*% mass*[a]	*Metal stoichiometry*[a]	*% mass*[b]	*Metal stoichiometry*[b]
**Cu**	10.354 (10.344)	6.03 (6.02)	10.41	6.05
**Ni**	6.359 (6.339)	4.01 (3.99)	6.39	4.03
**Ag**	11.598 (11.577)	3.98 (3.97)	11.63	3.98
**Na**	1.233 (1.229)	1.98 (1.98)	–	–

[a]Solid samples were digested with 0.5 mL of HNO_3 69% at 60°C for 4 hours followed by the addition of 0.5 mL of HCl 37% and digestion 80°C for 1 hour. Values in parentheses correspond to ICP-MS measurements for **3** after catalytic experiments. [b]SEM/EDX measurements were carried out on solid policrystalline samples of **2** and **3**.

Table S2. Summary of Crystallographic Data for **2** and **3.**

Compound	**2**	**3**
Formula	$C_{78}H_{162}Ag_4Cu_6N_{12}Ni_4O_{87}$	$C_{78}H_{156}Ag_4Cu_6N_{12}Na_2Ni_4O_{84}$
M (g mol^{-1})	3707.75	3699.68
λ (Å)	0.6889	0.6889
Crystal system	tetragonal	tetragonal
Space group	$P4/mmm$	$P4/mmm$
a (Å)	35.7978(7)	36.037(6)
c (Å)	15.0057(3)	15.311(2)
V (Å^3)	19229.5(8)	19884(7)
Z	4	4
ρ_{calc} (g cm^{-3})	1.281	1.236
μ (mm^{-1})	1.507	1.460
T (K)	90	90
θ range for data collection (°)	0.80- 23.27	0.79- 20.77
Completeness to θ_{max}	100% to θ = 23.273°	98.6% to θ = 20.773°
Measured reflections	152867	90965
Unique reflections (Rint)	7686 (0.0931)	5730(0.2125)
Observed reflections [$I > 2\sigma(I)$]	5325	3335
Goof	1.273	1.937
R^a [$I > 2\sigma(I)$] (all data)	0.1220 (0.1594)	0.1562 (0.2124)
wR^b [$I > 2\sigma(I)$] (all data)	0.3424 (0.3686)	0.3565 (0.3749)
CCDC	2155455	2155456

$^aR = \sum(|F_o| - |F_c|)/\sum|F_o|$. $^bwR = [\sum w(|F_o| - |F_c|)^2/\sum w|F_o|^2]^{1/2}$.

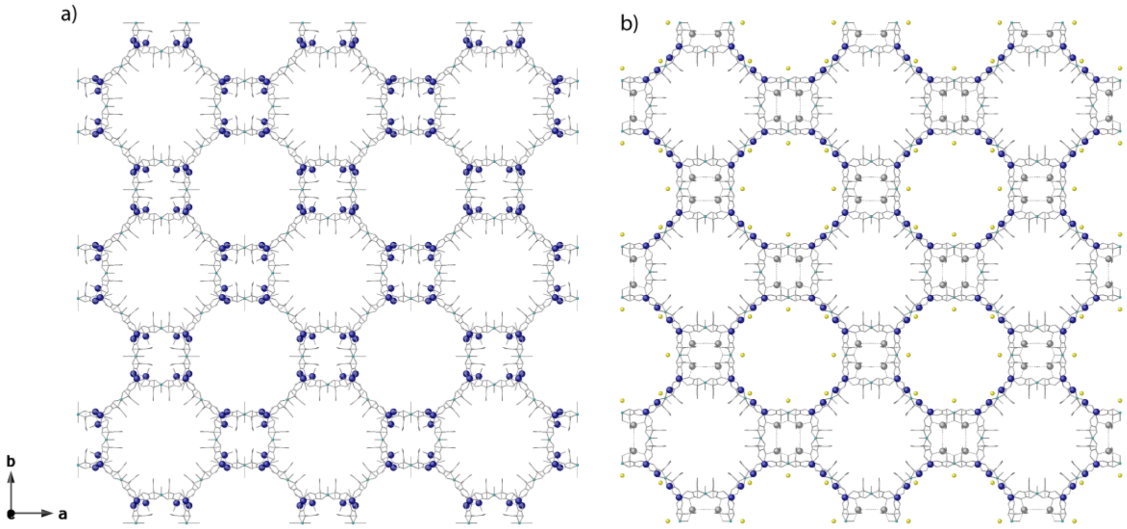

Figure S1. Perspective view along *c* crystallographic axis of crystal structures of **2** (a) and **3** (b) presenting channels filled by Ag^+ complexes (**2**) or Ag^0_2 NCs and Ag^+ ions (confined in square pores) (**3**). Lattice water molecules and hydrogen atoms have been omitted for clarity. Color scheme: Silver, blue sphere (octagonal pores of **2** and **3**) and grey spheres (Ag^+ ions not reduced in square pores in **3**); sodium, yellow spheres, ligands atoms and metal ions of the whole net have been depicted as grey sticks.

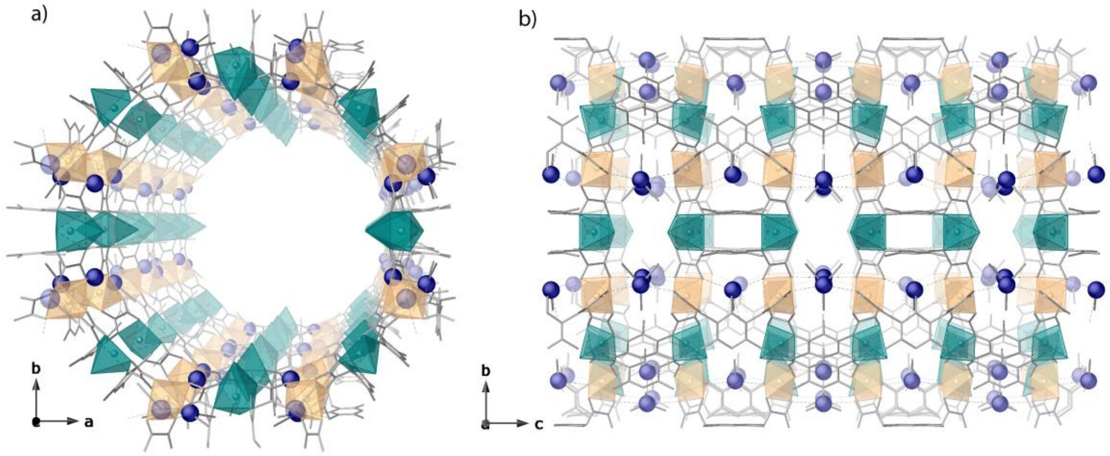

Figure S2. Details along *c* (a) and *a* (b) crystallographic axis of a single octagonal pore in **2**. Color scheme: Silver, blue sphere; Copper and nickel, cyan and orange polyhedral, respectively; ligands atoms of the whole net have been depicted as grey sticks.

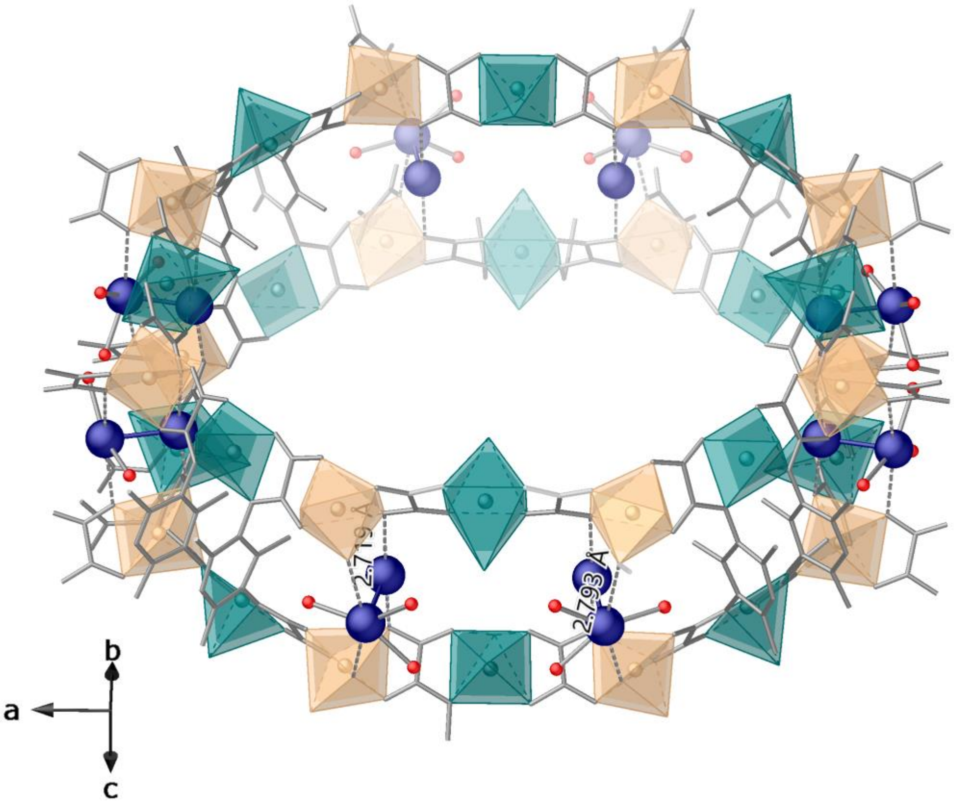

Figure S3. One single channel of **2** showing supramolecular interactions involving oxamate ligands of the network stabilizing Ag^+ dimers. Color scheme: Silver, blue sphere; Copper and nickel, cyan and orange polyhedral, respectively; ligands atoms of the whole net have been depicted as grey sticks. Modelled oxygen atoms (likely belonging to NO_3^- anions) surrounding Ag^+ ions are depicted as red spheres.

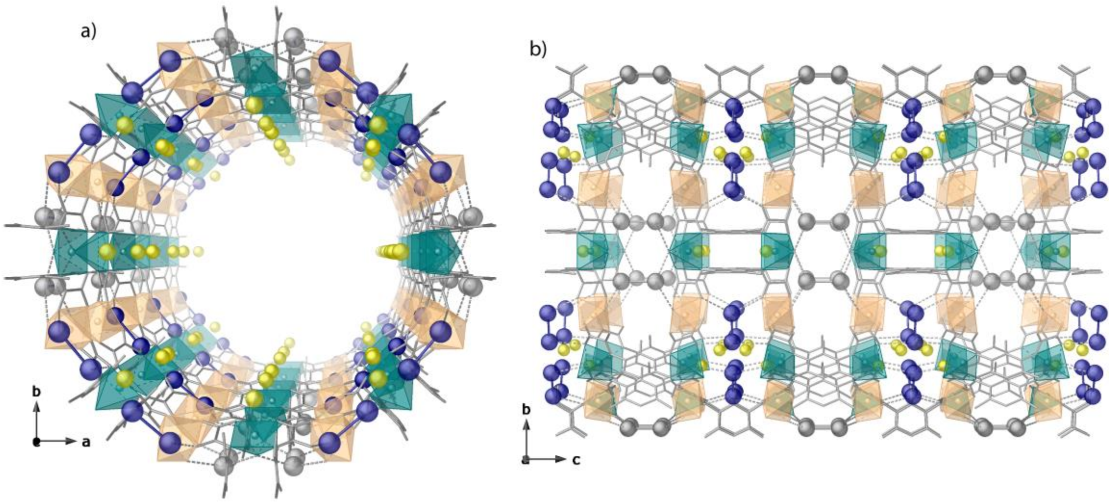

Figure S4 Details along *c* (a) and *a* (b) crystallographic axis of a single octagonal pore in **3**. Color scheme: Silver, blue sphere; Sodium, yellow spheres. Copper and nickel, cyan and orange polyhedral, respectively; ligands atoms of the whole net have been depicted as grey sticks.

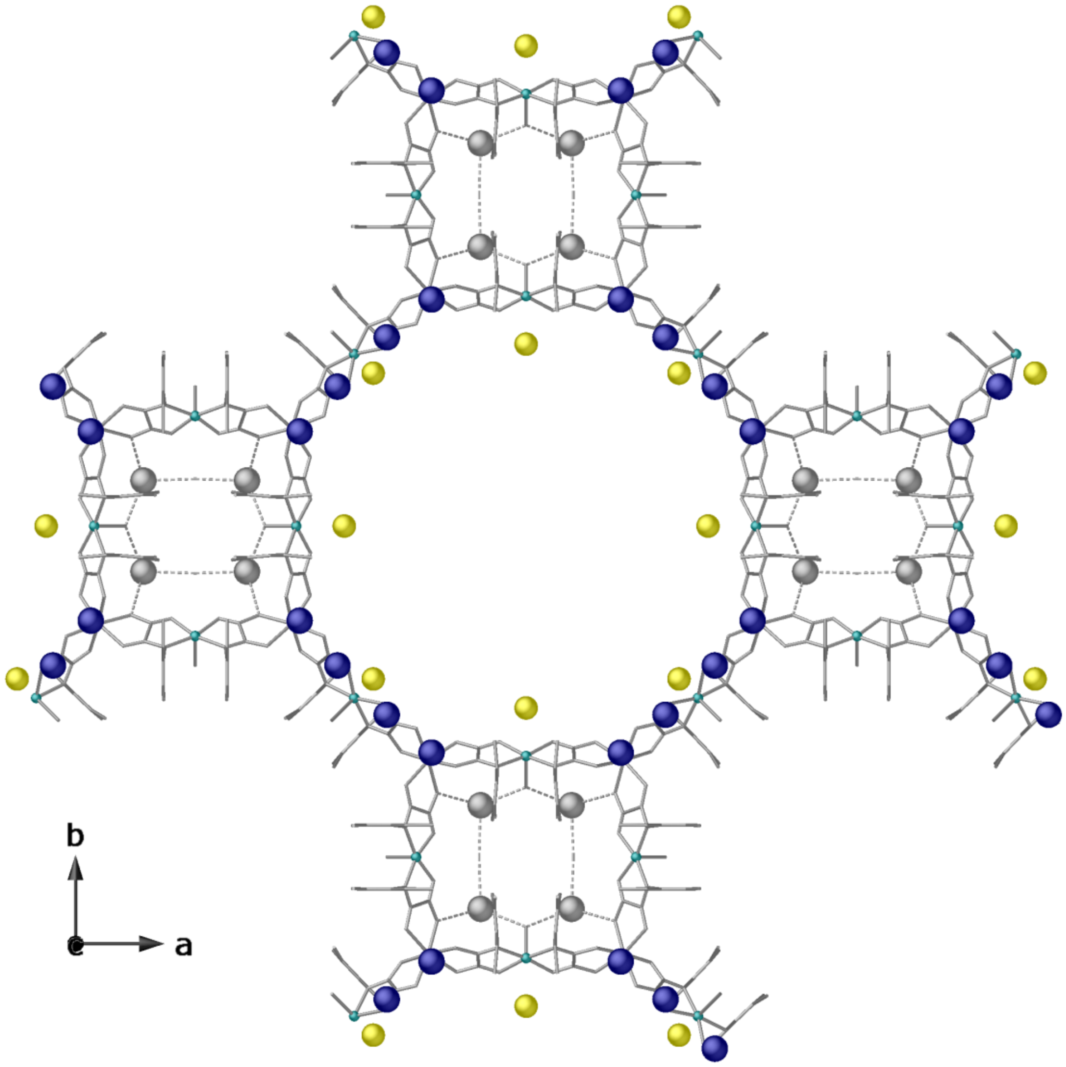

Figure S5. Details along *c* crystallographic axis of a portion of crystal structure of **3** showing disposition of Ag^+ ions (grey spheres) residing in poorer accessible small square pores. Color scheme: Silver, blue and grey spheres (Ag^+ ions not reduced in square pores); sodium, yellow spheres. Copper and nickel, cyan and orange polyhedral, respectively; ligands atoms of the whole net have been depicted as grey sticks.

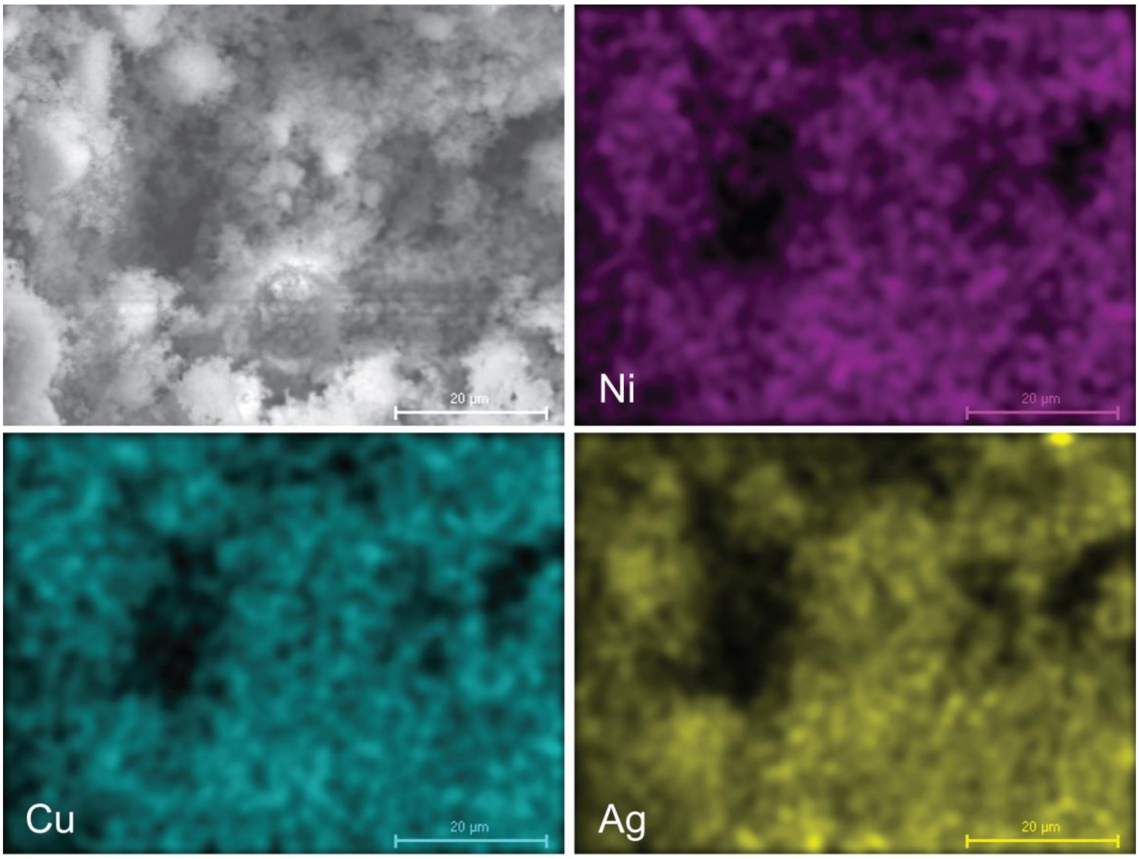

Figure S6. Backscattered SEM image of **2** and the corresponding EDX elemental mapping for Cu (cyan), Ni (magenta) and Ag (yellow) elements. The backscattering detector highlights the MOF particles as brighter areas due to crystalline MOF structure and to the presence of heavier atoms in the MOF than in the polymer matrix.

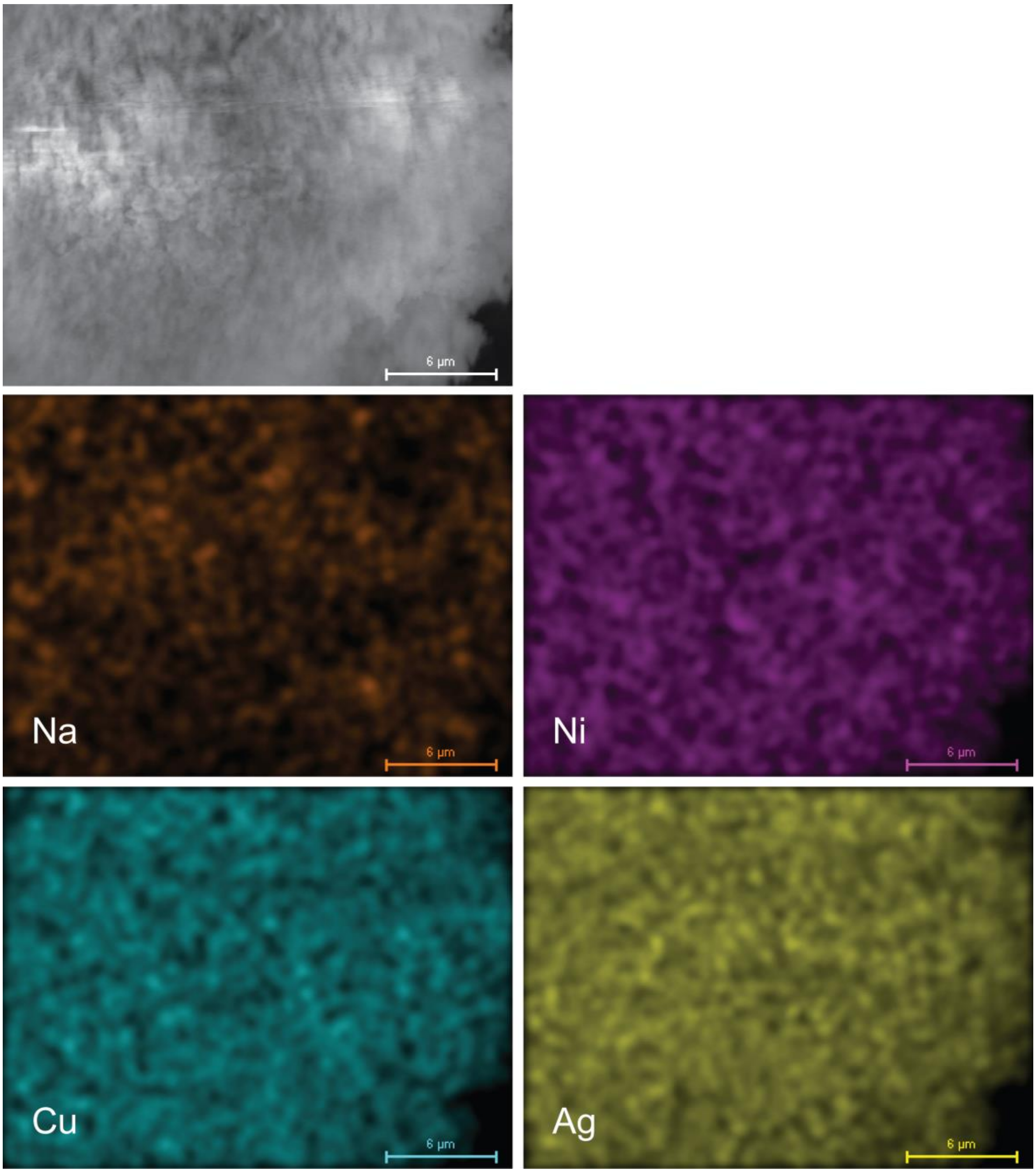

Figure S7. Backscattered SEM image of **3** and the corresponding EDX elemental mapping for Cu (cyan), Na (orange), Ni (magenta) and Ag (yellow) elements. The backscattering detector highlights the MOF particles as brighter areas due to crystalline MOF structure and to the presence of heavier atoms in the MOF than in the polymer matrix.

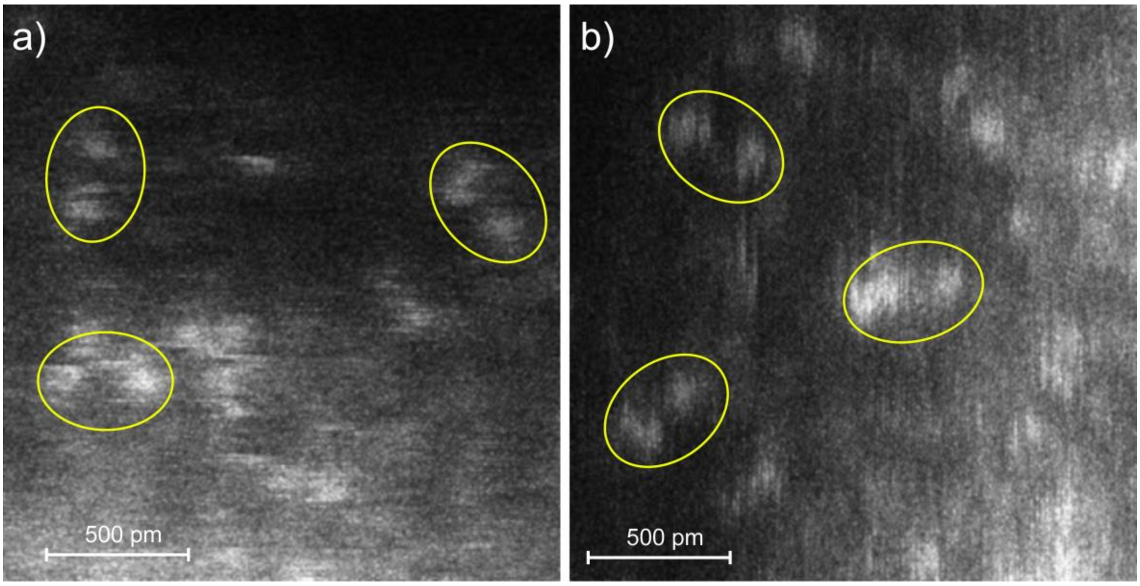

Figure S8. AC-HAADF-STEM images of MOF **3** showing the presence of both Ag single atoms and Ag_2 dimers (yellow circles).

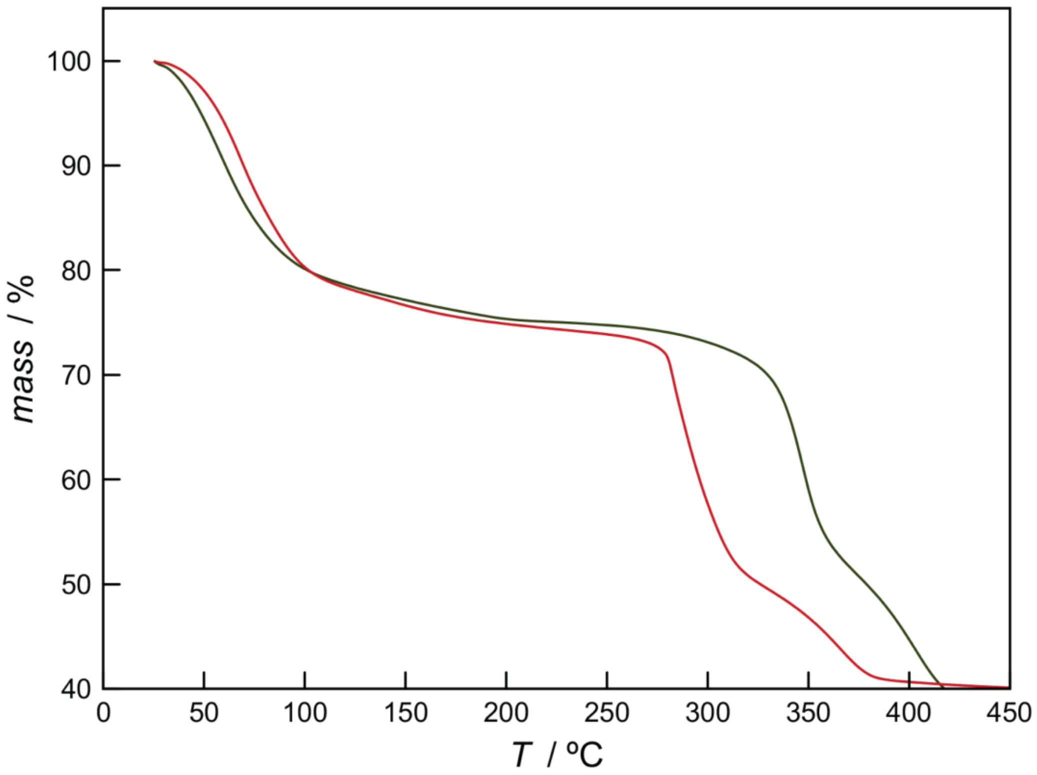

Figure S9. Thermo-Gravimetric Analyses (TGA) of **2** (red) and **3** (green) under a dry N_2 atmosphere.

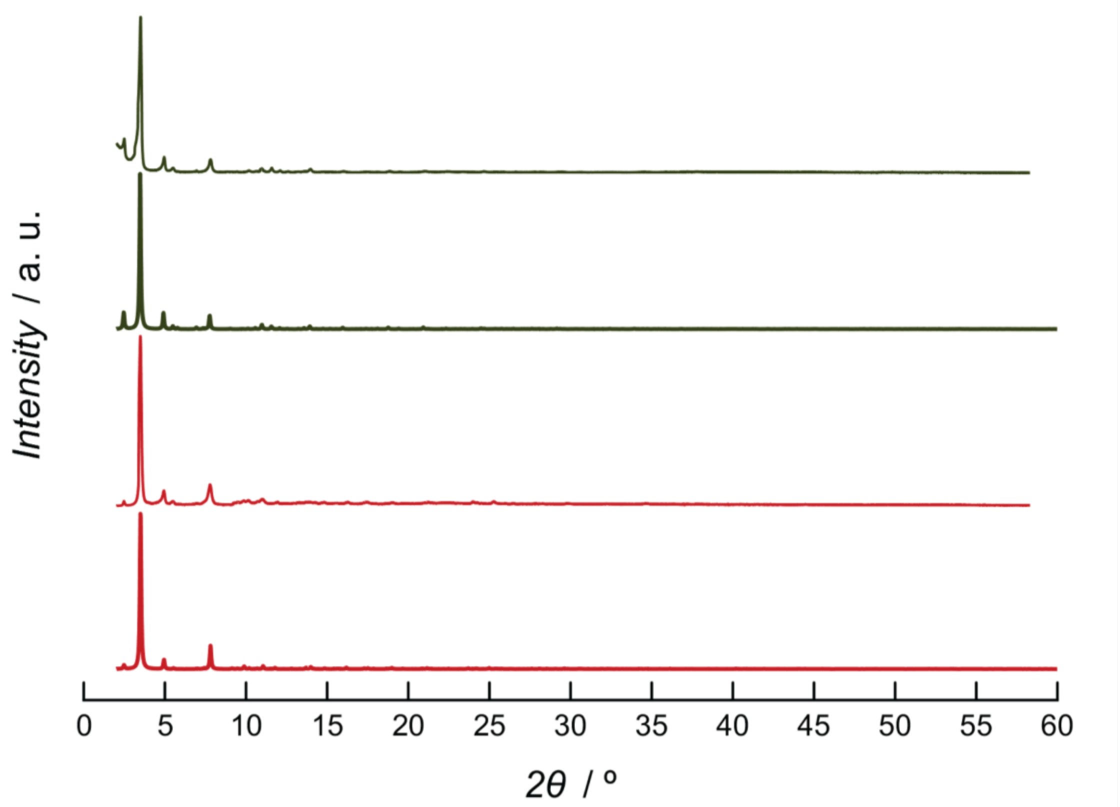

Figure S10. Theoretical (bold lines) and experimental (solid lines) PXRD pattern profiles of **2** (red) and **3** (green) in the 2θ range 2–60°.

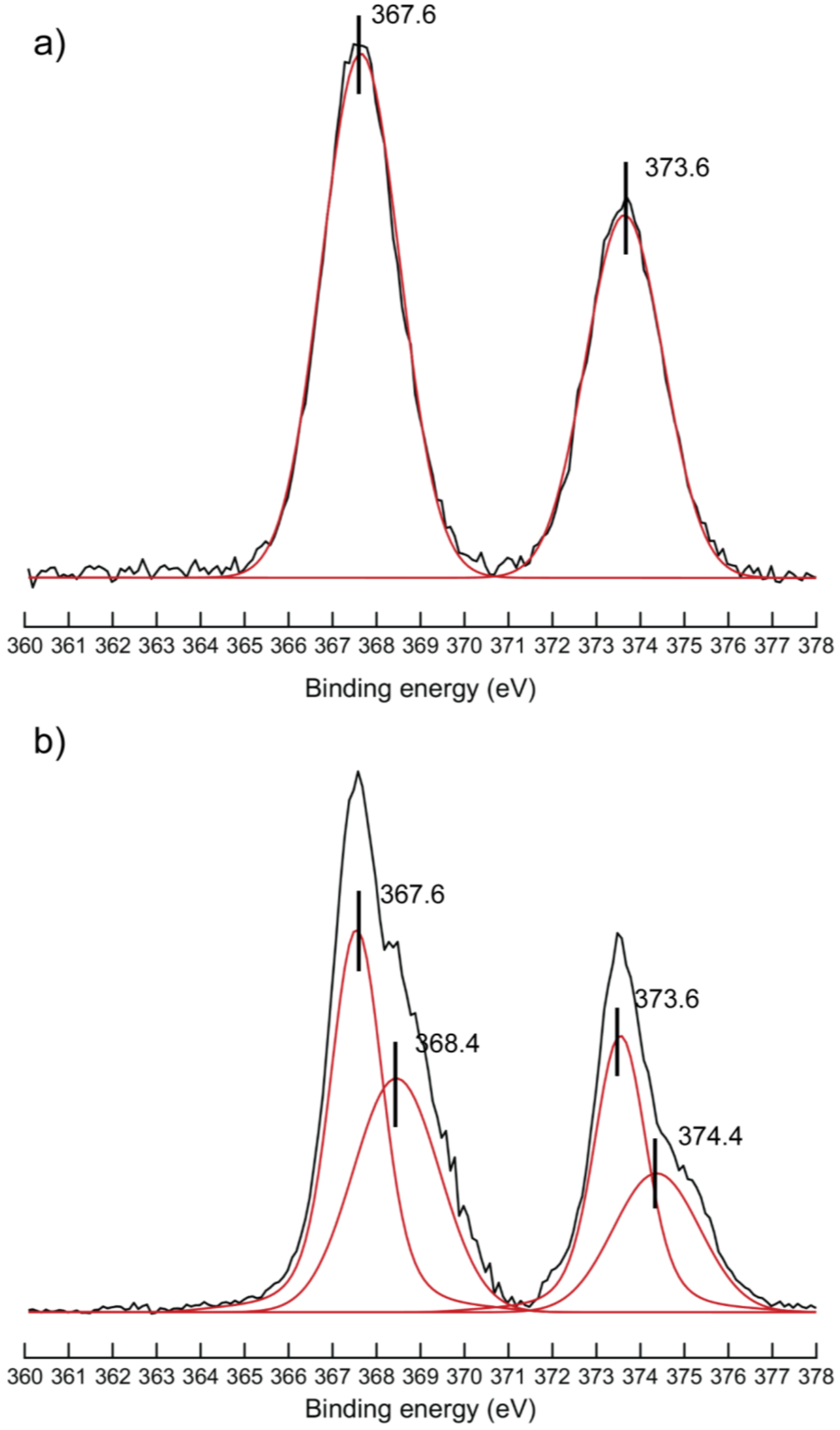

Figure S11. X–ray photoelectron spectroscopy (XPS) of **2** (a) and **3** (b).

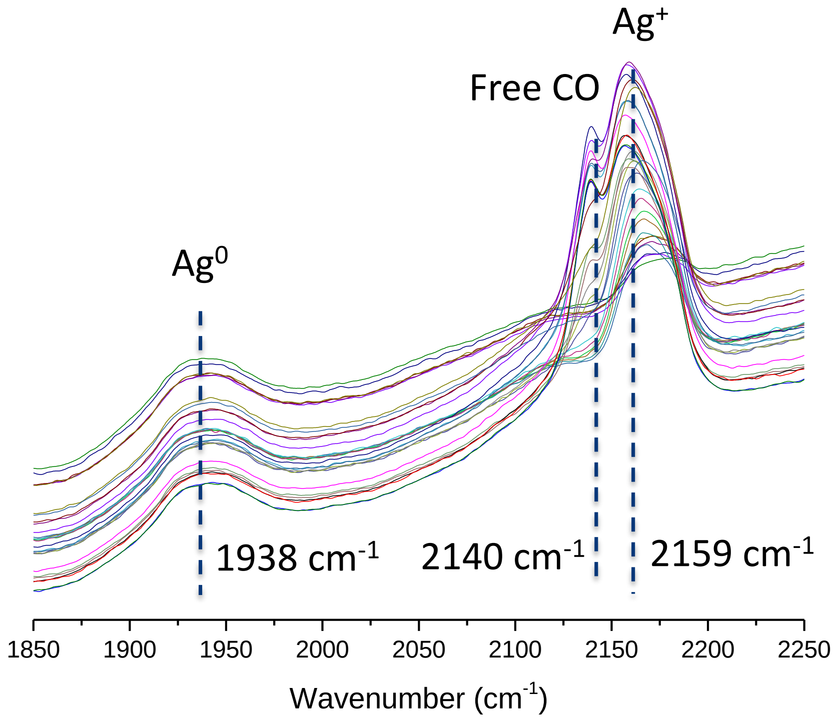

Figure S12. CO-probe diffuse reflectance infrared fourier transform spectroscopy (DRIFTS) of MOF **3**, run at 77 K with different CO doses at later desorption.

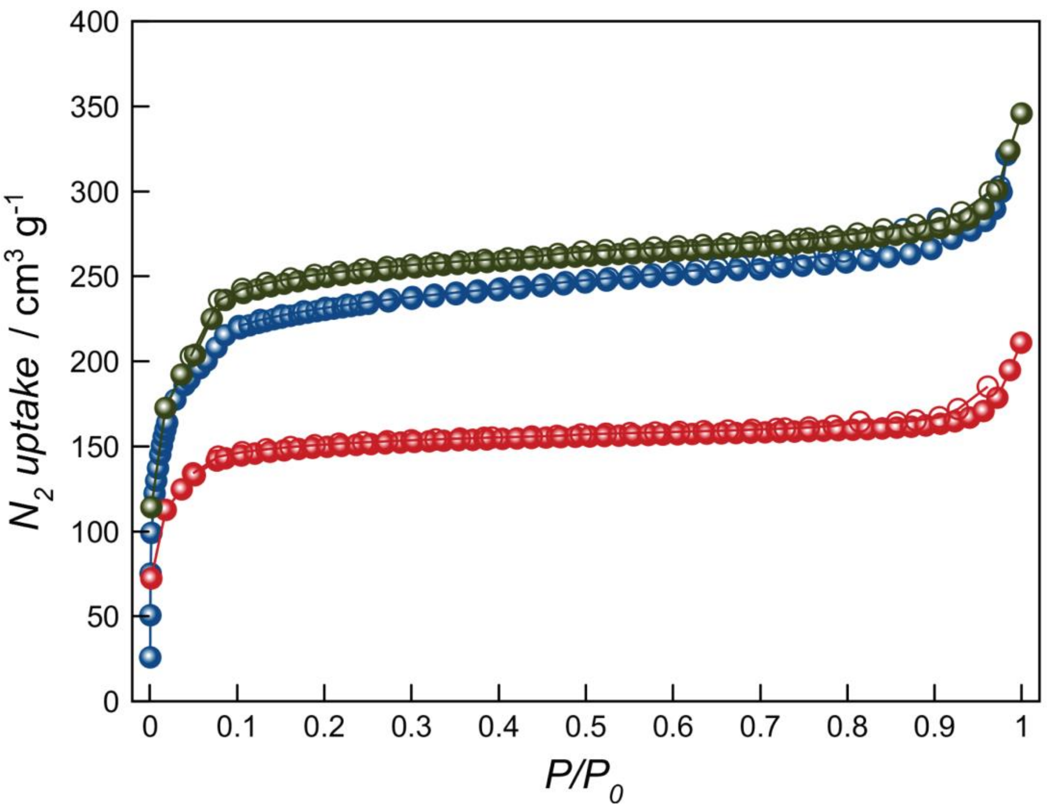

Figure S13. N_2 sorption (filled circles) and desorption (empty circles) isotherms for the activated compounds **1** (blue), **2** (red) and **3** (green) at 77 K.

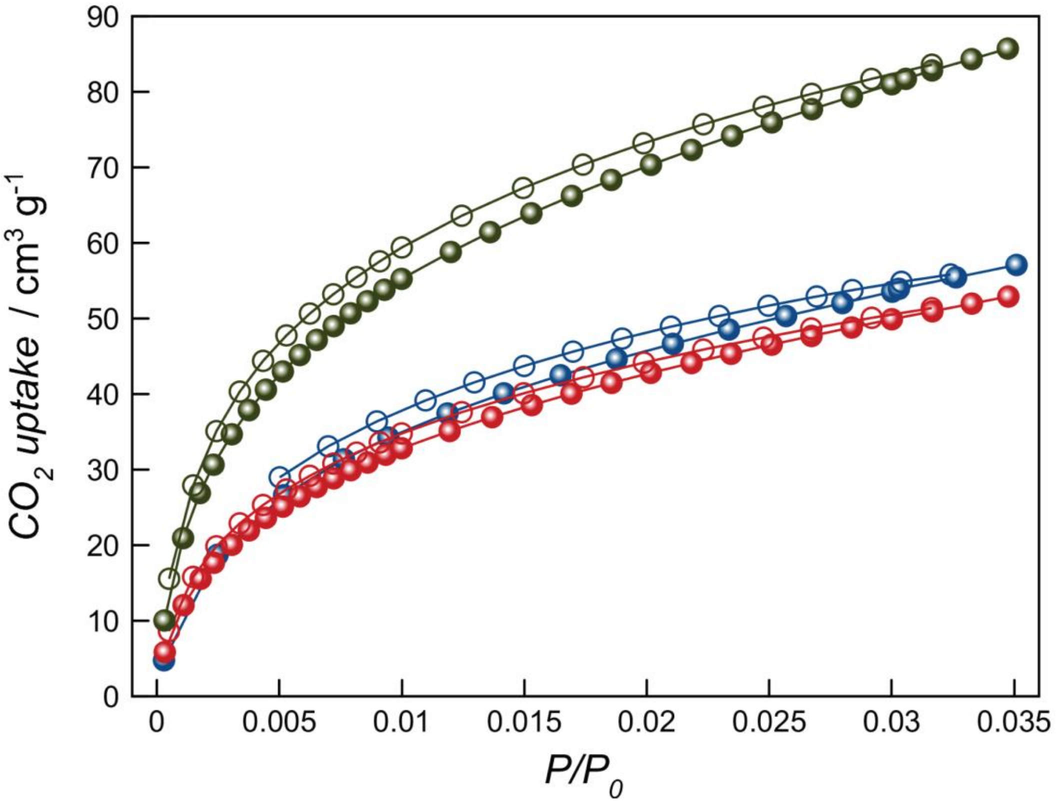

Figure S14. CO_2 sorption (filled circles) and desorption (empty circles) isotherms for the activated compounds **1** (blue), **2** (red) and **3** (green) at 273 K.

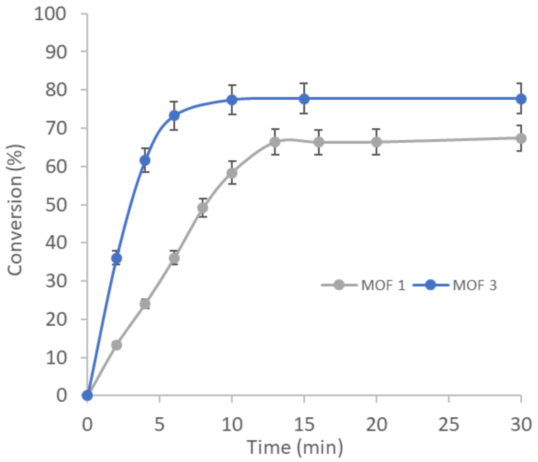

Figure S15. Kinetics for the Buchner ring expansion reaction between toluene **4** and ethyl diazoacetate **5** catalyzed by MOFs **1** and **3**. Error bars account for a 5% uncertainty. **5** is added at once.

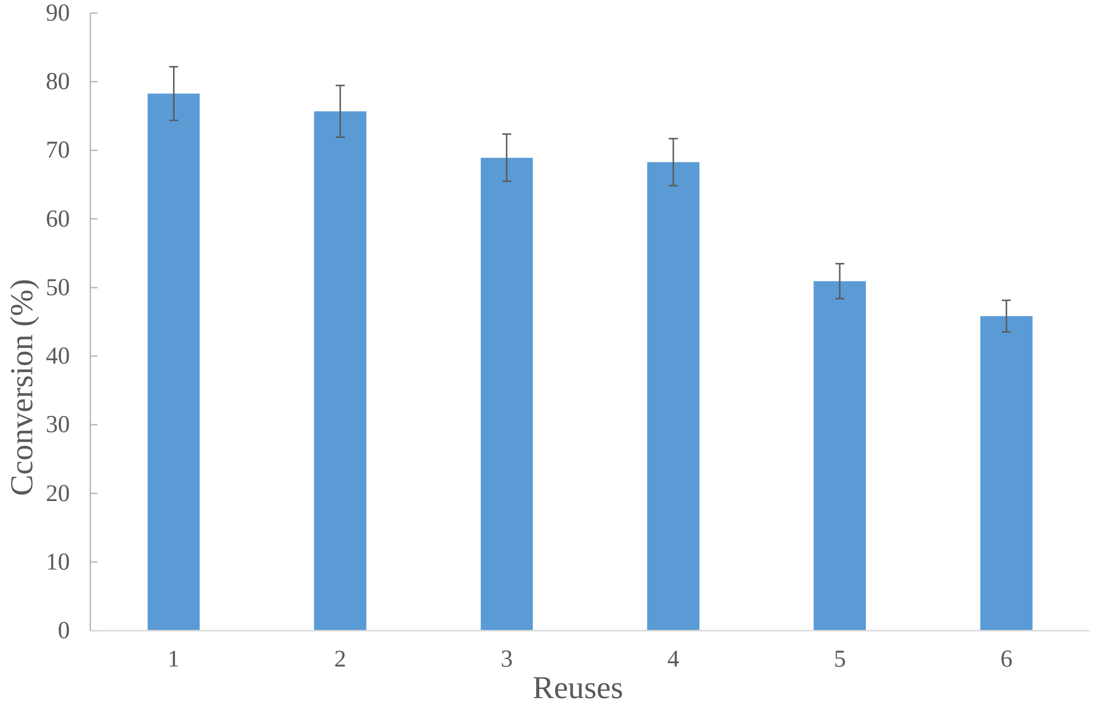

Figure S16. Catalytic reuses for the Buchner ring expansion reaction between toluene **4** and ethyl diazoacetate **5** catalyzed by MOF **3** under the reaction conditions indicated in the main text. **5** is added at once. MOF **3** is recovered from the reaction mixture by centrifugation, washed with dichloromethane, and reused. Error bars account for 5% uncertainty.

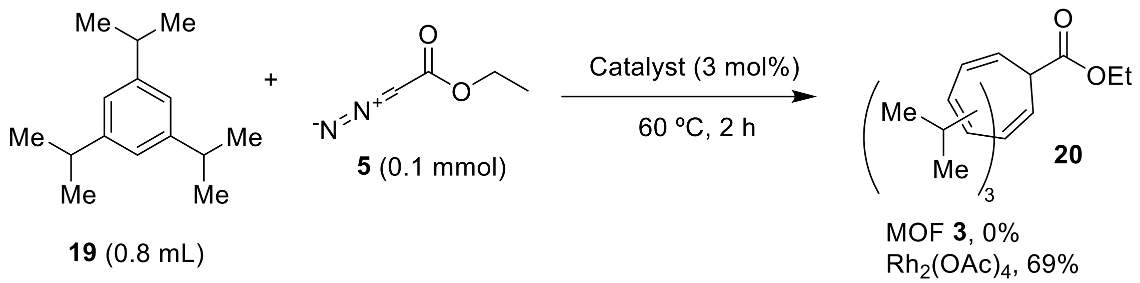

Figure S17. Buchner ring expansion reaction between mesitylene **18** and ethyl diazoacetate **5** catalyzed by either MOF **3** or $Rh_2(OAc)_4$ salt (3 mol%) under the indicated reaction conditions. **5** is added at once.

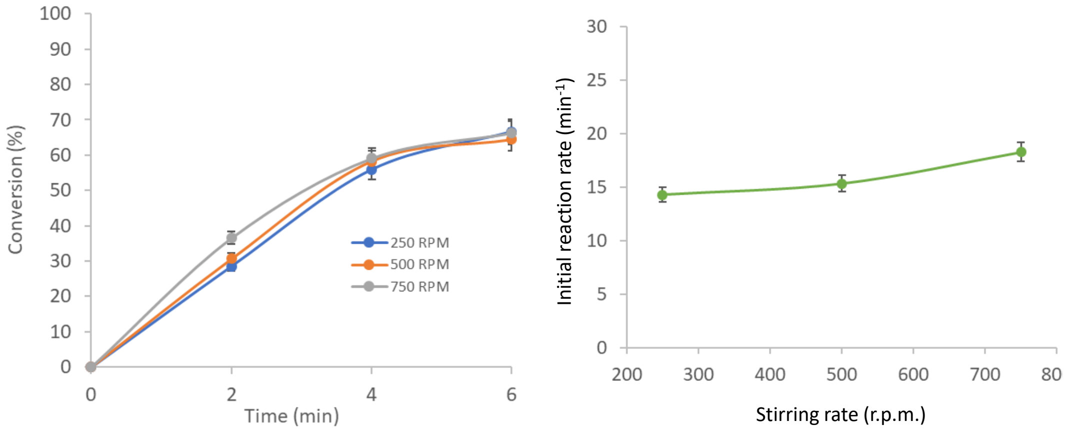

Figure S18. Left: Kinetics for the Buchner ring expansion reaction between toluene **4** and ethyl diazoacetate **5** catalyzed by MOF **3** under increasing stirring speeds. Error bars account for a 5% uncertainty. **5** is added at once. Right: the corresponding initial rate-stirring rate correlation.

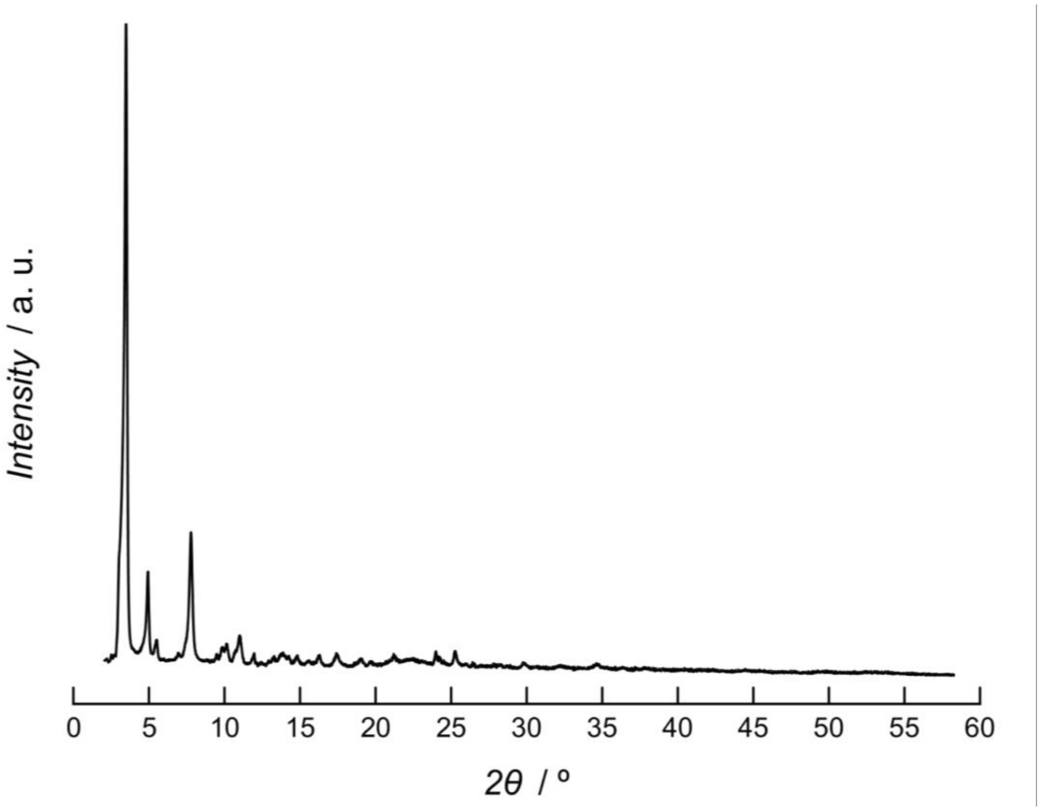

Figure S19. Experimental PXRD pattern profile of **3**, in the 2θ range 2–60°, after catalytic experiments.

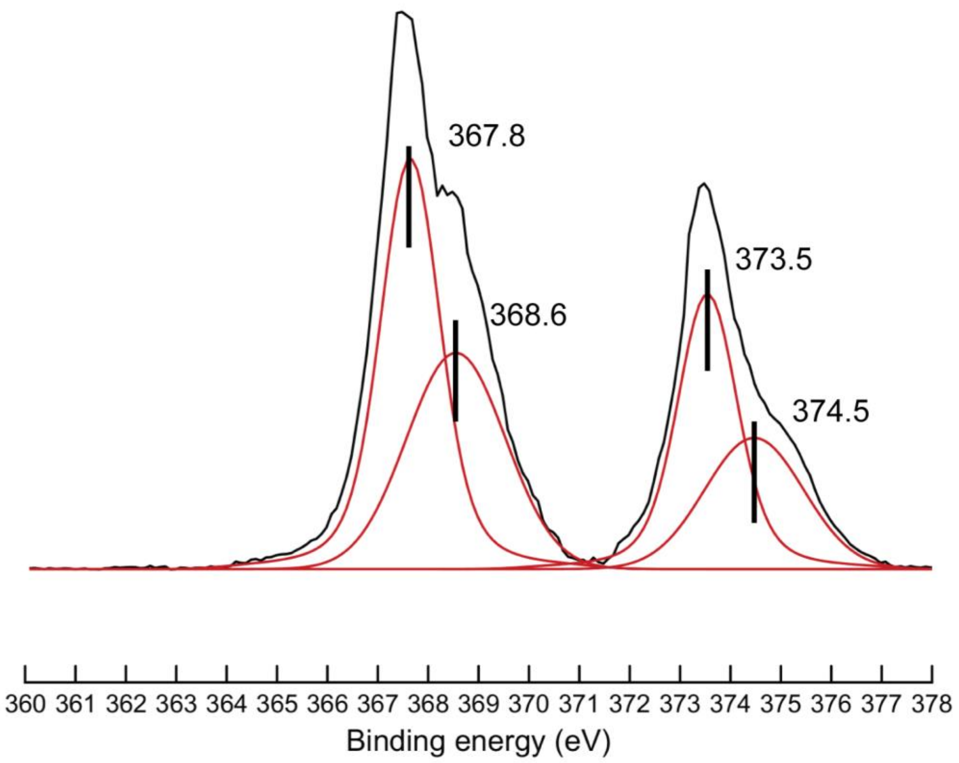

Figure S20. X–ray photoelectron spectroscopy (XPS) of **3** after catalytic experiments.

Characterization of the products.

Ethyl methylcyclohepta–2,4,6–trienecarboxylate **6**. ^{1}H NMR (δ, ppm; *J*, Hz; diagnostic signals underlined): *2–methyl:* 6.50–5.50 (5xC*H*, 5H), 4.18 (C*H*2, 2H, q, *J*= 7.0), 2.75 (C*H*, 1H, d, *J*= 6.7), 2.20 (C*H*3, 3H, s), 1.26 (C*H*3, 3H, t, *J*= 7.0); *3– methyl:* 6.43–5.90 (3xC*H*, 3H), 4.95–4.60 (2xC*H*, 2H), 4.10 (C*H*2, 2H, q, *J*= 7.0), 2.15 (C*H*, 1H, t, *J*= 5.0), 1.86 (C*H*3, 3H, s), 1.21 (C*H*3, 3H, t, *J*= 7.0); *4–methyl:* 6.45–5.90 (2xC*H*, 2H), 5.35 (3xC*H*, 3H, mult), 4.15 (C*H*2, 2H, q, *J*= 7.0), 2.45 (C*H*, 1H, t, *J*= 5.3), 1.98 (C*H*3, 3H, s), 1.22 (C*H*3, 3H, t, *J*= 7.0). ^{13}C NMR (δ, ppm; unless indicated, indistinguishable signals for each isomer): 174.0–170.0 (C), 140.0 (2xC *2–methyl*), 131.0–114.0 (CH), 68.0–60.5 (CH2), 49.0–40.0 (CH), 24.0–20.7 (CH3), 14.2–14.0 (CH3).

GC-MS copies:

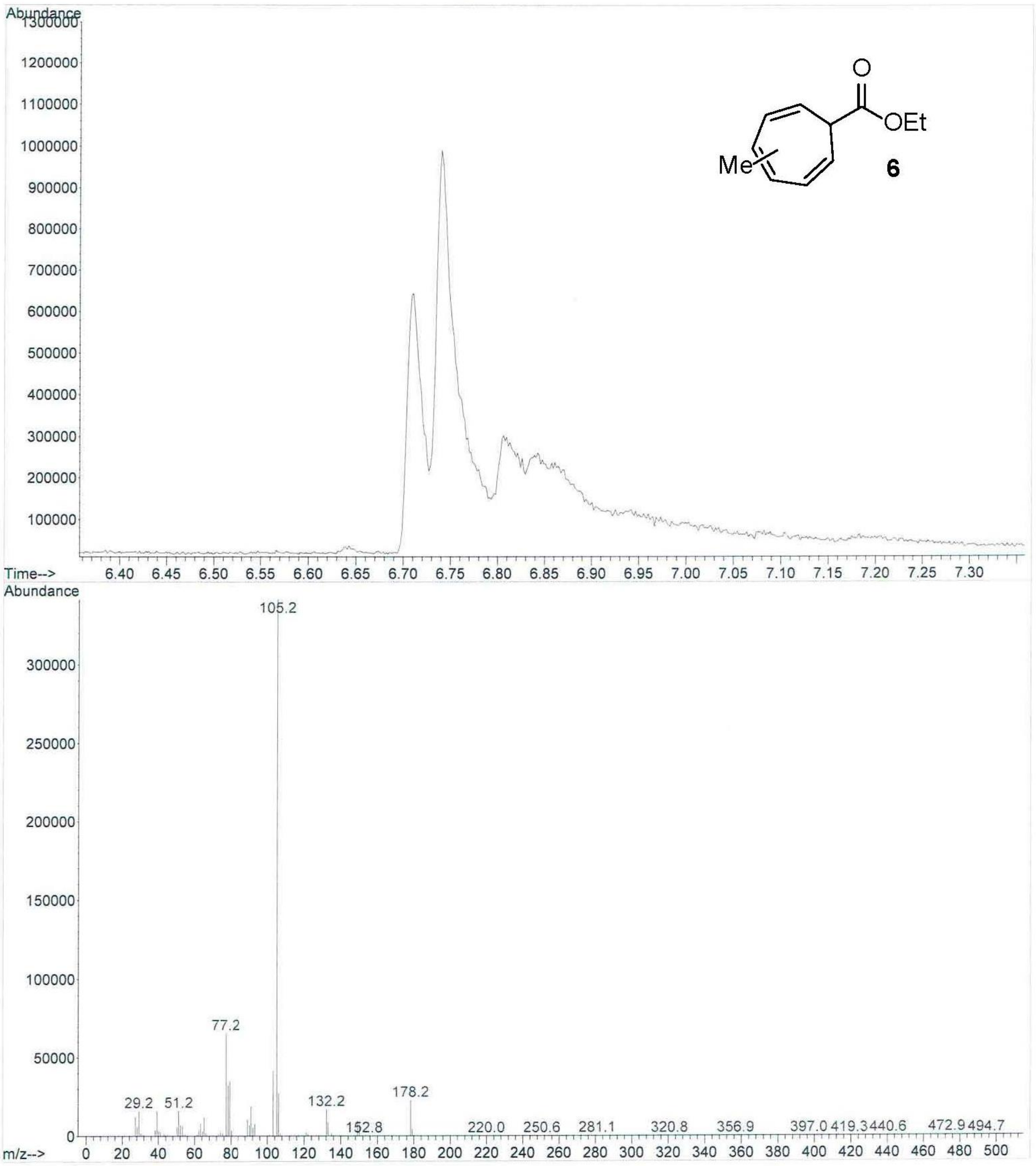

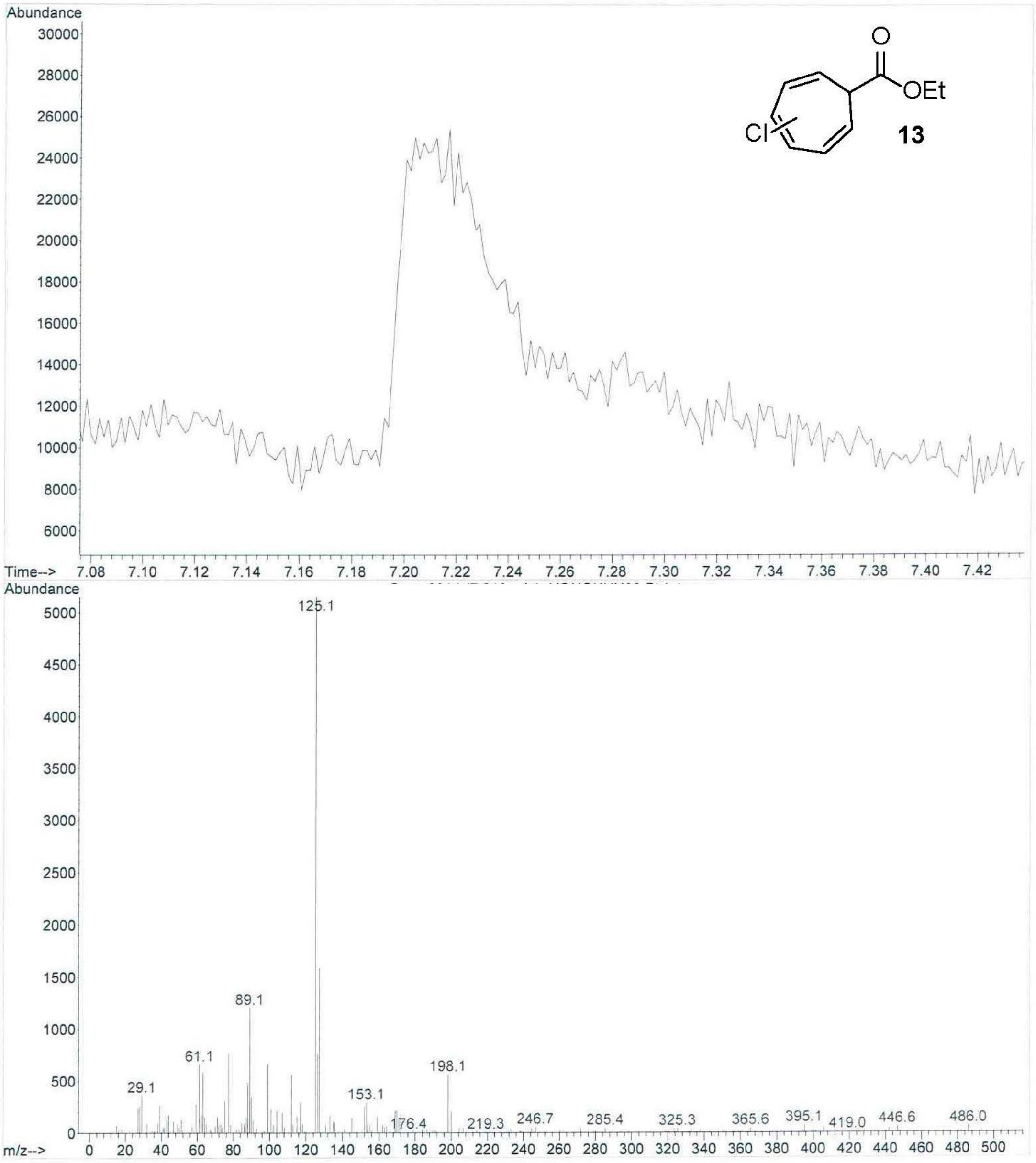
Abundance
Time-->
m/z-->
125.1
89.1
61.1
29.1
198.1
153.1
176.4
219.3
246.7
285.4
325.3
365.6
395.1
419.0
446.6
486.0
O
OEt
Cl
13

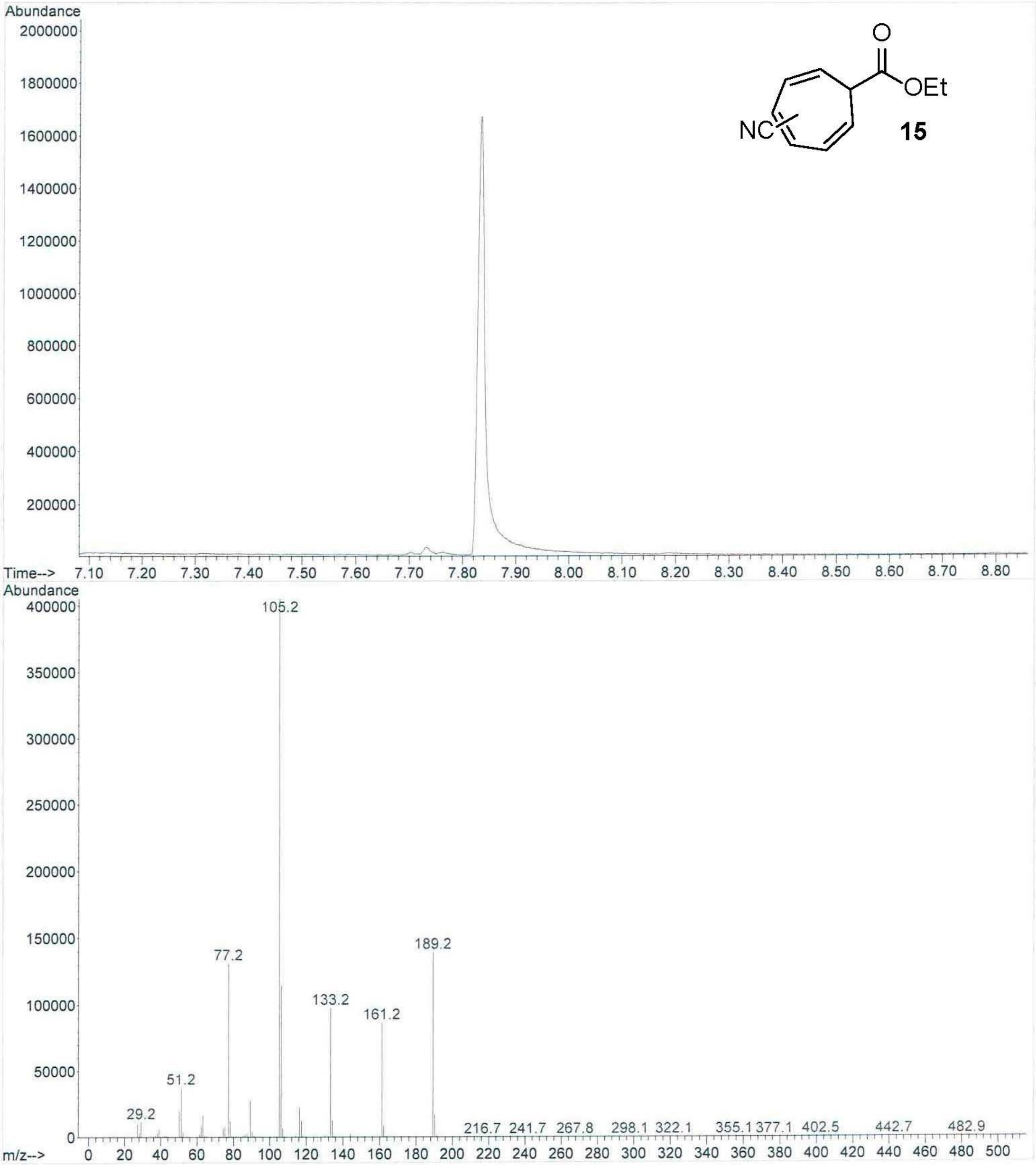
Abundance
2000000
1800000
1600000
1400000
1200000
1000000
800000
600000
400000
200000
Time--> 7.10 7.20 7.30 7.40 7.50 7.60 7.70 7.80 7.90 8.00 8.10 8.20 8.30 8.40 8.50 8.60 8.70 8.80
O
OEt
NC
15
Abundance
400000
350000
300000
250000
200000
150000
100000
50000
0
105.2
77.2
189.2
133.2
161.2
51.2
29.2
216.7 241.7 267.8 298.1 322.1 355.1 377.1 402.5 442.7 482.9
m/z--> 0 20 40 60 80 100 120 140 160 180 200 220 240 260 280 300 320 340 360 380 400 420 440 460 480 500

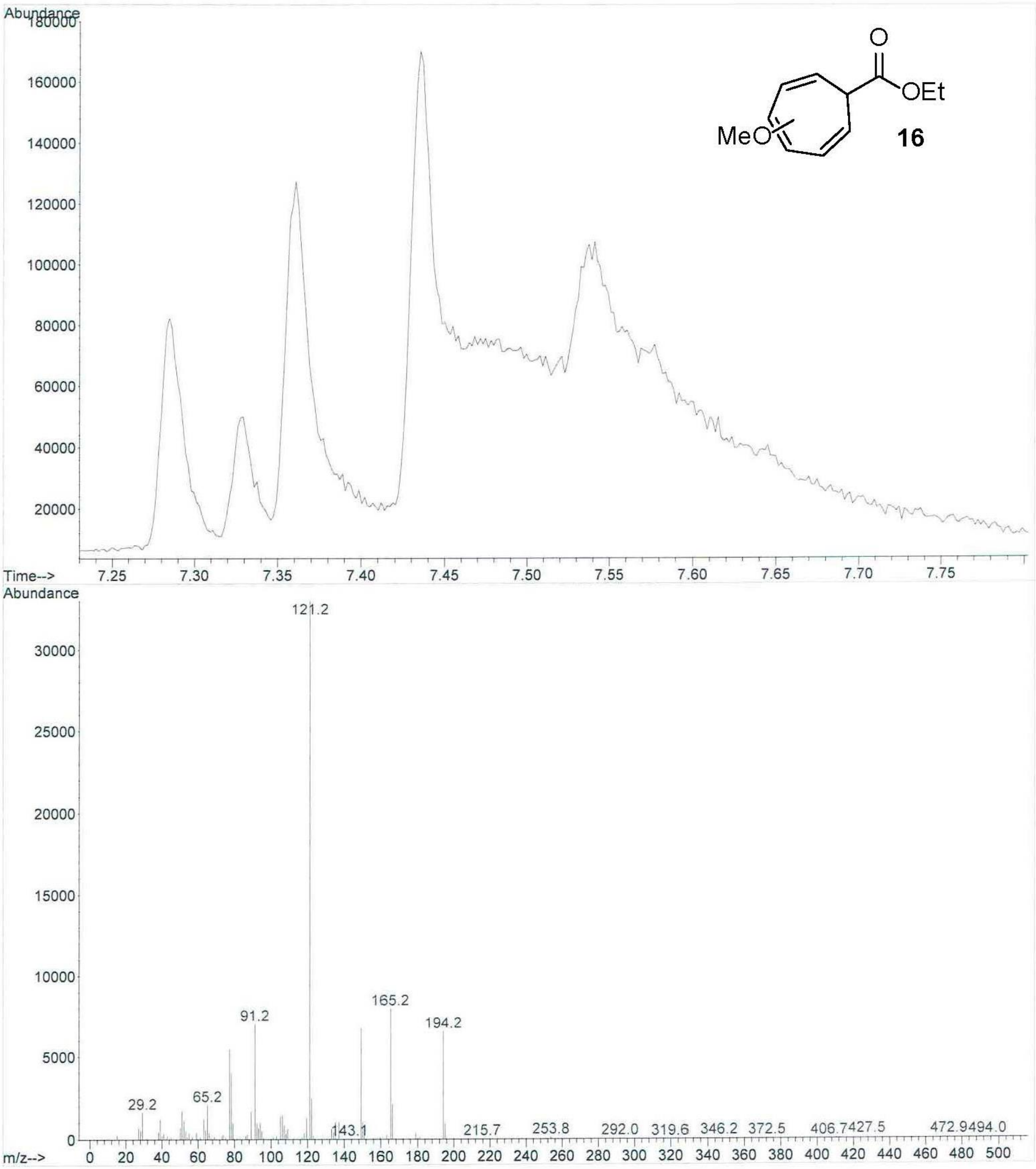
Abundance
Time-->
7.25 7.30 7.35 7.40 7.45 7.50 7.55 7.60 7.65 7.70 7.75
O
OEt
MeO
16
Abundance
121.2
165.2
194.2
91.2
65.2
29.2
143.1
215.7 253.8 292.0 319.6 346.2 372.5 406.7427.5 472.9494.0
m/z-->

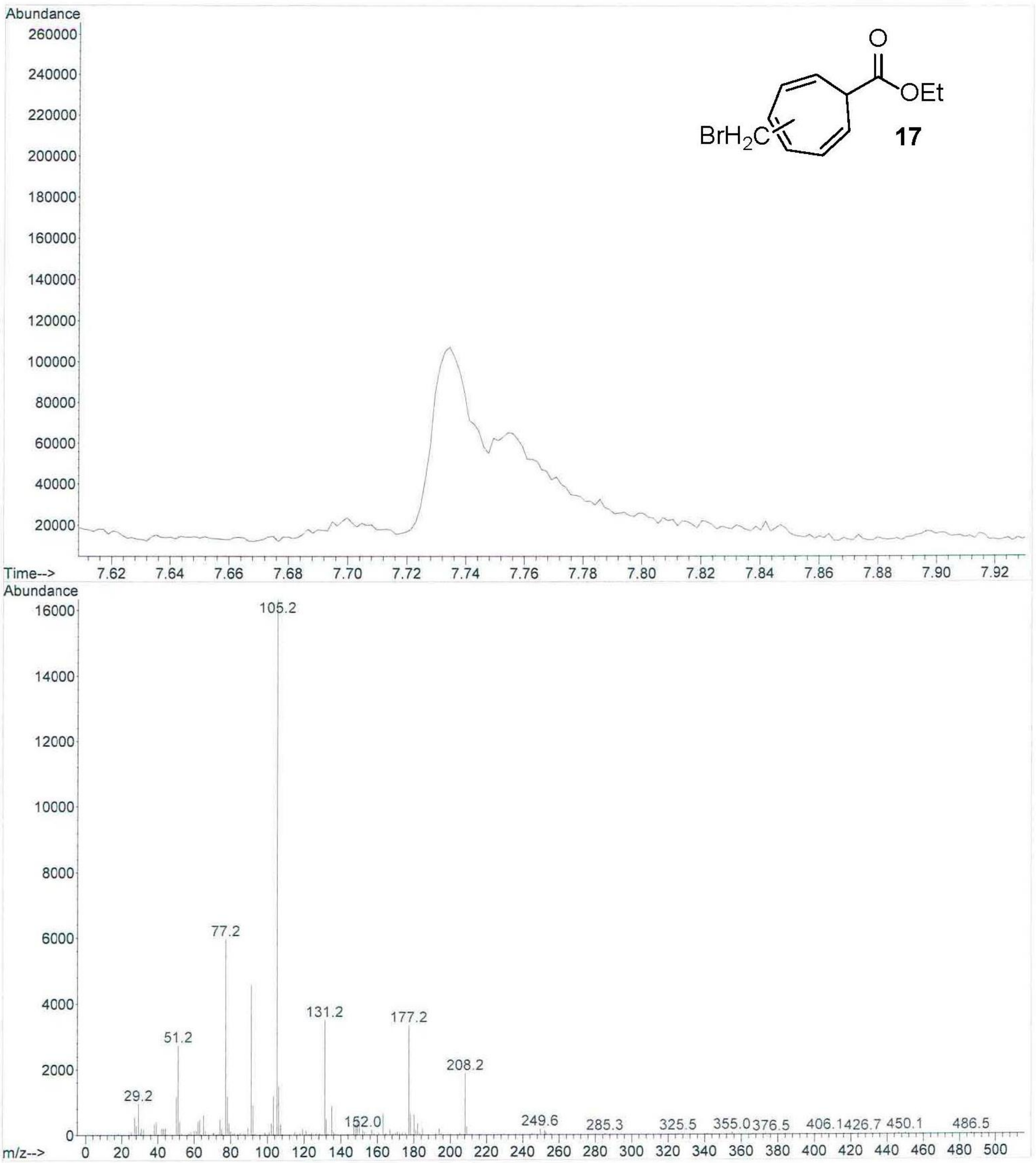

Abundance
BrH2C
OEt
O
17
Time-->
m/z-->
105.2
77.2
51.2
29.2
131.2
152.0
177.2
208.2
249.6
285.3
325.5
355.0
376.5
406.1
426.7
450.1
486.5

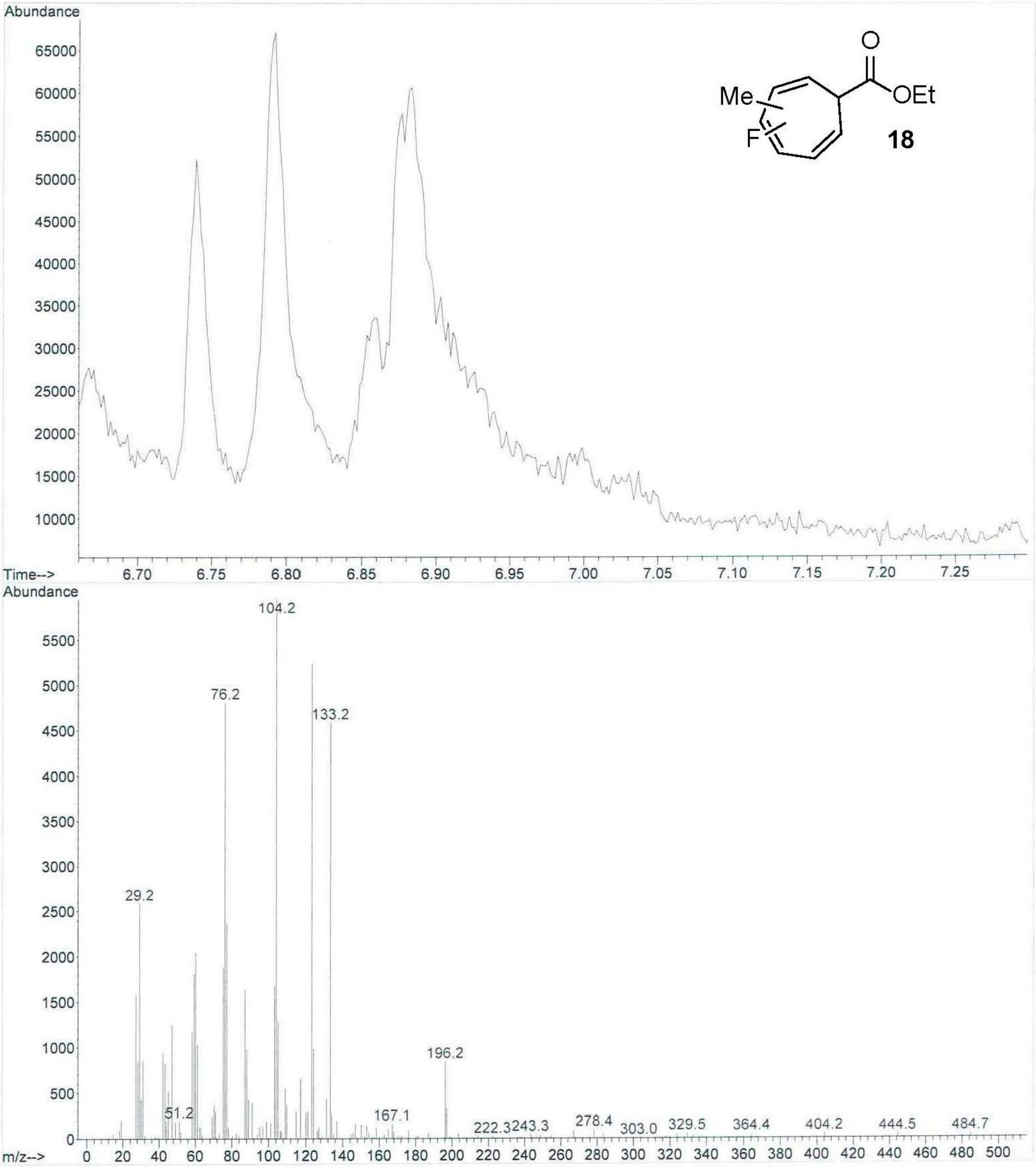
Abundance
Time-->
Abundance
m/z-->
104.2
76.2
133.2
29.2
51.2
196.2
167.1
222.3
243.3
278.4
303.0
329.5
364.4
404.2
444.5
484.7
Me
F
O
OEt
18
